# Supplementary material for: Reduced Breast and Ovarian Cancer Through Targeted Genetic Testing: Estimates Using the NEEMO Microsimulation Model
Source: Cancers (Basel). 2024 Dec 13;16(24):4165. doi: 10.3390/cancers16244165 (PMC11674464; doi:10.3390/cancers16244165)
Supplement: Supplementary file 1 [file cancers-16-04165-s001.zip › cancers-3288757-supplementary.pdf]

Supplemental Materials

Contents

1. Model diagrams 3
Figure S1. Population generation flowchart 3
Figure S2. Main simulation model sequence 3
Figure S3. Time cycle sequence 4
2. Population generation 5
2.1 Life history 5
2.2 Family size 5
2.3 Inter-family interactions 5
2.4 Late simulation entry for unborn family members 5
2.5 Inherited breast and ovarian cancer risk 5
Figure S4. Probability density of age at first birth by total parity 6
Figure S5. Probability density of birth intervals by total parity 7
3. Cancer pathology and mortality 8
3.1 Breast cancer natural history 8
3.2 Breast cancer mortality 8
3.3 Ovarian cancer mortality 8
4. Model input tables 9
Table S1. Number of births (parity) by age group at model entry 9
Table S2. Prevalence of pathogenic/likely pathogenic variants for the input population 9
Table S3. Cancer risk by genotype and age 10
Table S4. Hazard ratio per standard deviation for the polygenic risk score 10
Table S5. Tumour pathology for breast and ovarian cancer 11
Table S6. Breast cancer-specific mortality 12
Table S7. Ovarian cancer-specific mortality 13
Figure S6. Assumptions related to predictive testing in relatives. 14
Table S8. Clinical effectiveness of risk management strategies 15
Table S9. Uptake and adherence to cancer risk management strategies 16
5. Model assumptions 17
6. Population validation 19
Figure S7. Parity by age group in observed versus simulated population. 19

Figure S8. Distribution of birth ages by child order compared to observed Australian population data. 20
Figure S9. Family history of breast cancer in first degree relatives by gene. 21
Table S10. Validation of prevalence of pathogenic variants by personal history of cancer. 22
7. Cancer outcomes validation 23
Figure S10. Cumulative breast cancer incidence by gene. 23
Figure S11. Cumulative ovarian cancer incidence by gene. 24
Figure S12. Distribution of ovarian cancer histology by age group. 25
Figure S13. Distribution of ovarian cancer grade and stage 25
Figure S14. Distribution of breast cancer pathology by mode of detection 26
Figure S15. Relative survival after ovarian cancer by histology. 27
Figure S16. Relative survival after ovarian cancer by age group. 28
Figure S17. Relative survival after breast cancer by age. 29
8. Genetic testing validation 30
Figure S18. Simulated referral rates for genetic counselling and genetic testing after a cancer diagnosis. 30
Figure S19. Validation of referral rates for young-onset breast cancer and high-grade serous ovarian cancer. 31
Figure S20. Uptake of predictive testing in relatives compared to observed data. 32
Figure S21. Validation of risk-reducing surgery uptake in BRCA1/2 pathogenic/likely pathogenic variant carriers 33
9. Model outcomes 34
Figure S22. Breast cancer survival after a cancer diagnosis in relatives of pathogenic/likely pathogenic variant carriers. 34
Figure S23. Ovarian cancer survival after a cancer diagnosis in relatives of pathogenic/likely pathogenic variant carriers. 35
Table S11. Genetic outcomes in the relatives of probands who carry a P/LP variant, including age groups 36
Table S12. Clinical outcomes in relatives for the sensitivity analysis 39
References 40

1. Model diagrams

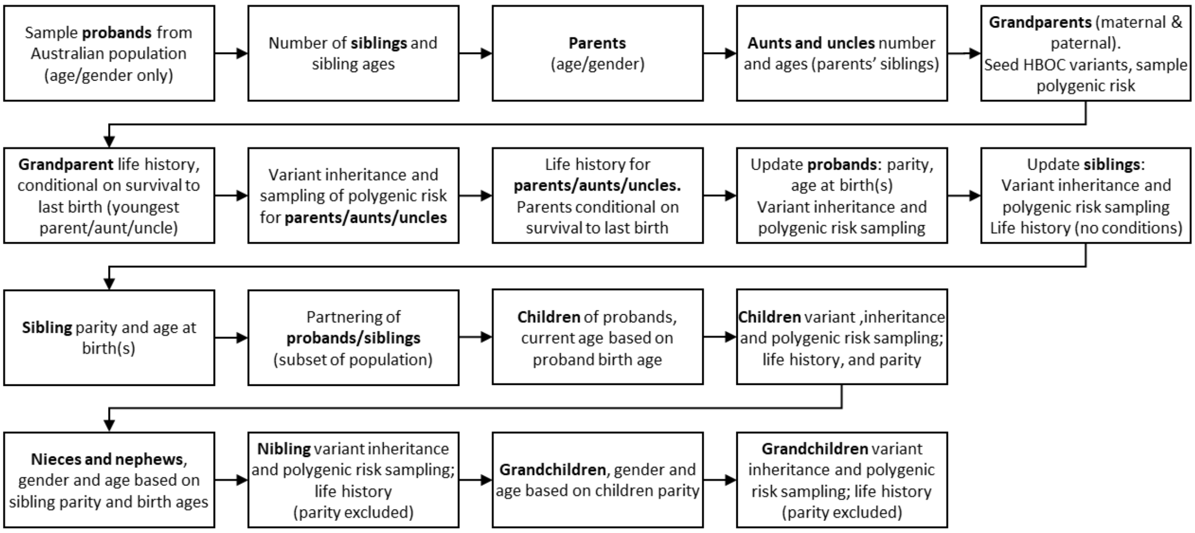

Figure S1. Population generation flowchart

The proband refers to the index women, who serve as the centre of each family unit (see Figure S6 for an example pedigree).

Abbreviations: HBOC: hereditary breast and ovarian cancer.

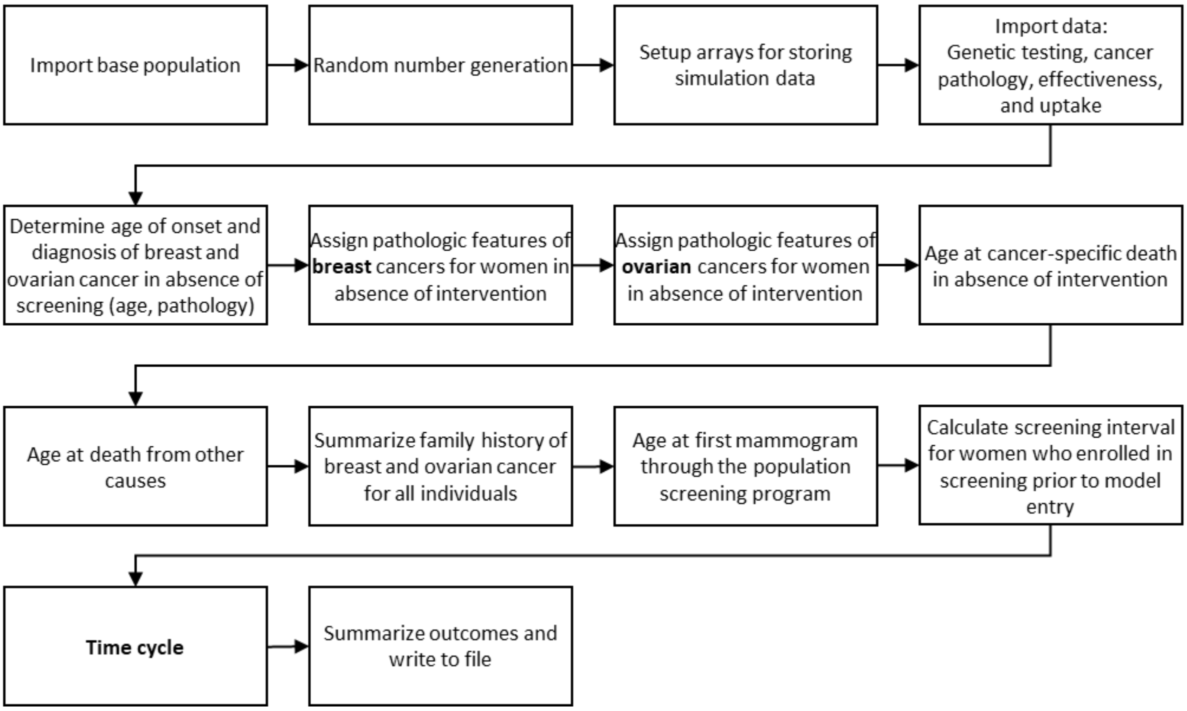

Figure S2. Main simulation model sequence

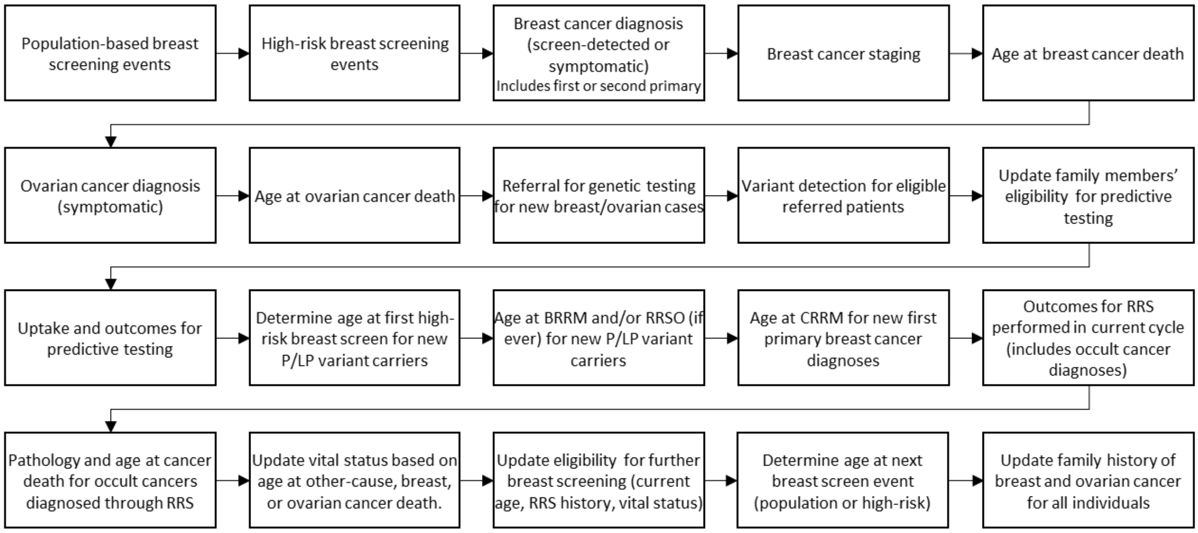

Figure S3. Time cycle sequence

Abbreviations: BRRM: bilateral risk-reducing mastectomy; CRRM: contralateral risk-reducing mastectomy; P/LP: pathogenic/likely pathogenic; RRS: risk-reducing surgery; RRSO: risk-reducing salpingo-oophorectomy.

2. Population generation

2.1 Life history

Life histories prior to the age at simulation entry for parents and grandparents are conditional on surviving to the age at their last birth. As breast and ovarian cancer treatment often affects fertility, it is assumed mothers and grandmothers of the proband would not be diagnosed with cancer prior to the age at last birth. For all other relatives, life histories are simulated prior to determining parity and birth ages so are not conditional on these events.

2.2 Family size

The number of first- and second-degree relatives is determined using age-specific cumulative distributions for parity (number of births, Table S1). Parity is assigned to all probands, parents, grandparents, siblings, and children in the model ranging from nulliparity (no births) to nine. Parents logically have a minimum parity of one (the proband), as do the grandparents (mother or father of the proband). The age at first birth is conditional on the total parity, meaning a higher number of offspring is associated with a younger age at first birth (Figure S4Figure S4). For subsequent births, the age at birth is calculated using birth intervals sampled from a log-normal distribution (Figure S5).

Each proband can have between 0-9 siblings. For proband siblings, the age of the first (eldest) sibling is relative to the age of the proband (constrained between -20 and +20 years), and sampled from cumulative distributions according to the total number of siblings. The age of subsequently born siblings is then assigned using a birth interval as described above.

2.3 Inter-family interactions

Linkage between unrelated family units is possible through proband-sibling and sibling-sibling partnerships. For example, a proband can have offspring with an unrelated sibling from another proband's family unit, so any resulting children or grandchildren from this pairing would not be duplicated, and would remain linked to both families.

2.4 Late simulation entry for unborn family members

An additional option is available to enable inclusion of unborn relatives who can enter the model after commencement of the main simulation. For example, if probands enter the main simulation at a young age such as 20-24 years, they are unlikely to have children or grandchildren upon entry. Opting for inclusion of unborn relatives allows for these future offspring to also be simulated. If this option is selected, then parity for all women aged younger than 55 years at the start of the simulation is assigned according to the 50-54 years age group estimates, rather than the age-specific estimates provided in Table S1. This means if a woman enters the model at age 20 years, and her age at first birth is at age 34 years, then her first-born child enters the main simulation aged 0 years during the 14th cycle.

2.5 Inherited breast and ovarian cancer risk

Single P/LP variants are inherited top-down from the grandparents through to subsequent generations. Direct offspring have a 50% chance of inheriting a P/LP variant. In the case where one parent is not directly simulated (such as the father of a proband's children), the probability of inheriting a P/LP variant from the non-simulated parent is relative to the

original prevalence estimate for each gene (0.25 for two-degrees separation from grandparents, 0.125 for three-degrees separation).

Individuals are allocated polygenic risks for breast and ovarian cancer sampled from the standard normal distribution. The polygenic risk is passed to offspring by calculating the mean of the parental polygenic risk, and sampling from a normal distribution with this new mean and a standard deviation of 0.7. Polygenic risk scores are ranked within the population, and then individuals are assigned a percentile for their polygenic risk.

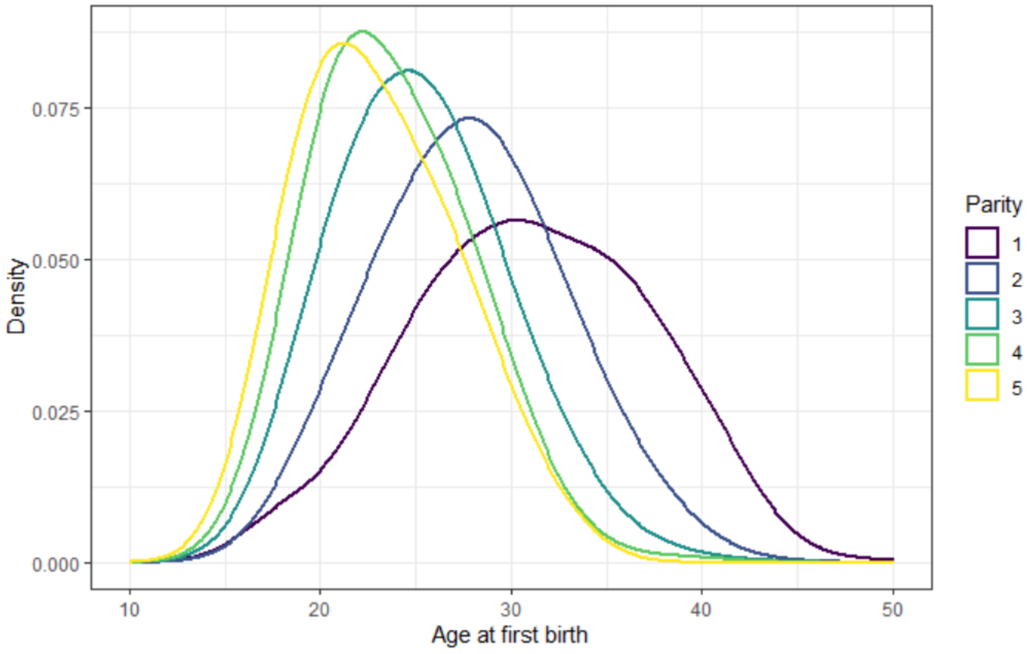

Figure S4. Probability density of age at first birth by total parity

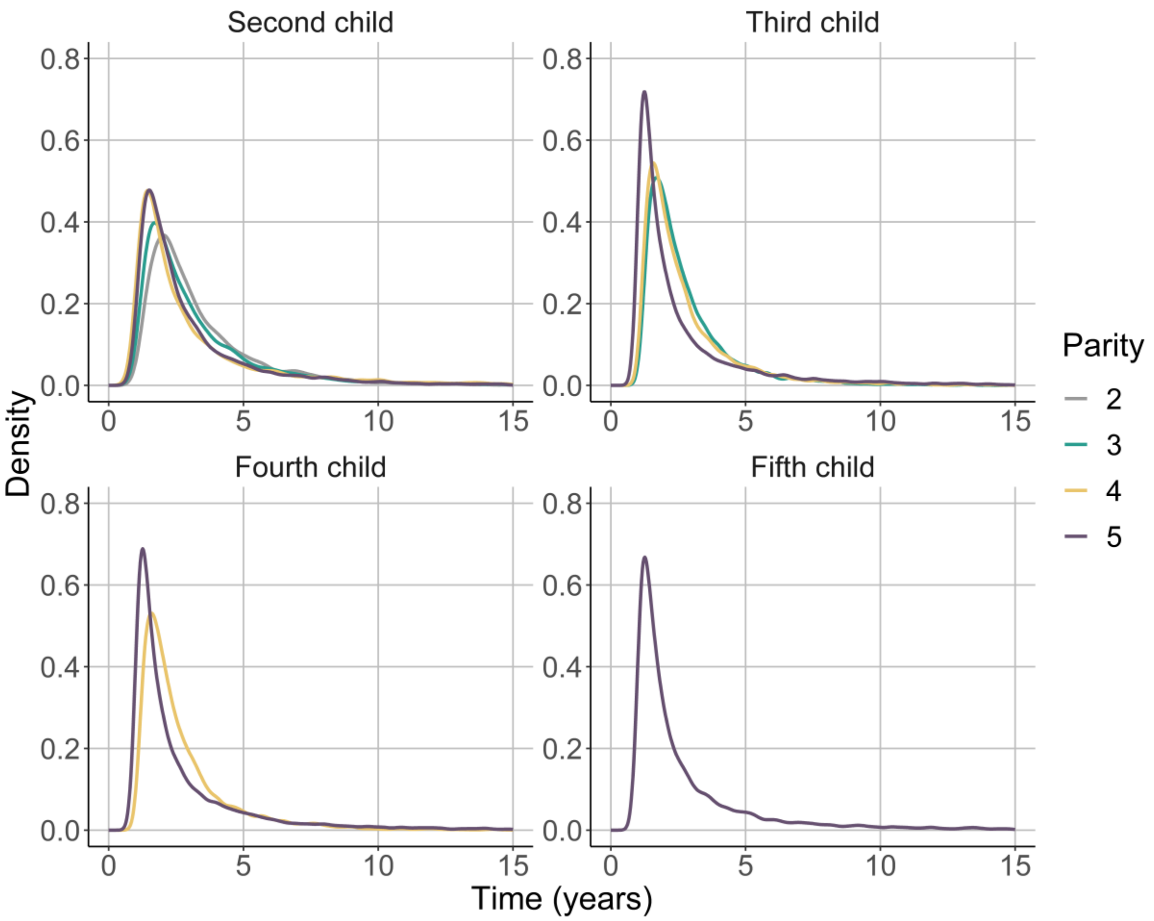

Figure S5. Probability density of birth intervals by total parity

3. Cancer pathology and mortality

3.1 Breast cancer natural history

Parameters for breast cancer pathology are described in Table S5. Carriers of P/LP variants in *CHEK2* or *ATM* are assumed to have a similar distribution of grade and hormone receptor status as *BRCA2*, while *PALB2* and *RAD51C/D* have a slightly higher proportion of triple negative disease to reflect what is currently known about the natural history.[1] Estrogen receptor (ER) status is associated with both gene and age (<55 years at onset) in the model. Progesterone receptor (PR) is associated with ER, and human epidermal growth factor receptor 2 (HER2) with ER, gene and age (<55 years). Grade is dependent on ER and HER2 status, gene, and being <55 years of age at tumour onset. The preclinical sojourn time is sampled from an age- and grade-specific Weibull distribution with parameters determined by a calibration of an Australian population-based breast cancer model.[2]

Tumour size is divided into seven categories: (1) < 7mm, (2) 7-9mm, (3) 10-19mm, (4), 20-29mm), (5) 30-39mm, (6) 40-49mm, and (7) ≥50mm. Size is assigned depending on the mode of detection (surgery, screen-detected, or symptomatic), gene, grade (1/2 or 3), ER, HER2 and age at diagnosis. Nodal status (positive or negative) is assigned depending on mode of detection, P/LP variant, tumour size, grade, ER status, and age. Due to the non-linear relationship between nodal status and tumour size for cancers <7mm and ≥50mm these are included as separate binary indicators.[3] Whether a tumour has distant metastasis on diagnosis is dependent on detection mode, nodal status, and ER status.

3.2 Breast cancer mortality

Woman are specified to be at risk of breast cancer-specific death for ten years from the point of diagnosis of invasive breast cancer or DCIS. Women with screen-detected or occult cancer on risk-reducing surgery are assumed to survive at least until the cancer would originally have been symptomatically diagnosed to reduce lead time bias. A Cox proportional-hazards model was applied using SEER data, and included covariates for grade, tumour size, nodal status, metastasis, molecular subtype, and age at diagnosis (Table S6).

3.3 Ovarian cancer mortality

Ovarian cancer mortality is also applied for ten years from diagnosis. Due to the proportional-hazards assumption being violated, two survival models were used for the subgroups mucinous and non-mucinous ovarian cancers. Covariates included histological subtype (non-mucinous model only) grade, age at diagnosis, and summary stage (Table S7).

4. Model input tables

Table S1. Number of births (parity) by age group at model entry

Age at model entry	Nulliparous	1	2	3	4	5	6	7	8	9
15-19	0.9779	0.8571	1							
20-24	0.8882	0.6706	0.9506	0.9800	1					
25-29	0.6662	0.4595	0.8784	0.9662	0.9802	1				
30-34	0.3999	0.3267	0.7877	0.9383	0.9846	0.9982	1			
35-39	0.2926	0.2192	0.7076	0.9363	0.9849	0.9946	0.9989	1		
40-44	0.1958	0.1781	0.6511	0.9063	0.9645	0.9972	0.9991	1		
45-49	0.1705	0.1531	0.6046	0.8822	0.9596	0.9845	0.9974	0.9979	1	
50-54	0.1862	0.1354	0.5702	0.8714	0.9689	0.9914	0.9979	0.9979	0.9990	1
55-59	0.1492	0.1180	0.5626	0.8601	0.9676	0.9906	0.9948	0.9979	0.9990	1
60-64	0.1153	0.1009	0.5080	0.8216	0.9434	0.9865	0.9926	0.9963	0.9975	1
65-69	0.1010	0.0877	0.4800	0.8017	0.9431	0.9815	0.9926	0.9963	0.9975	1
70+	0.0925	0.0844	0.4128	0.7814	0.9431	0.9815	0.9926	0.9963	0.9975	1

Cumulative distributions are based on 11,084 family pedigrees from the Parkville Familial Cancer Centre.

Table S2. Prevalence of pathogenic/likely pathogenic variants for the input population

	Original	Adjusted (age 0)	Source
BRCA1	0.001	0.0018	
BRCA2	0.0025	0.0034	[4,5]
PALB2	0.0024	0.0029	
ATM	0.0005	0.0005	
CHEK2	0.0082	0.0083	
RAD51C	0.0004	0.0004	
RAD51D	0.0004	0.004	
BRIP1	0.0017	0.0017	

Due to the method of ascertainment the average age of the cohort was 62 years, meaning for highly penetrant genes (such as BRCA1/BRCA2) the P/LP variant prevalence was likely to be an underestimate. The prevalence was therefore adjusted to account for women who would be excluded from Lifepool due to already having developed breast or ovarian cancer. Prevalence at age zero for each gene was estimated using lifetables, based on published gene- and age-specific cancer risk estimates (see Table S3).

Table S3. Cancer risk by genotype and age

Age	Non-carrier	*BRCA1*	*BRCA2*	*PALB2*	*CHEK2*	*ATM*	*RAD51C*	*RAD51D*	*BRIP1*
Source	AIHW, calibration	[6]	[6]	[7]	[8]	[9]	[10]	[10]	[11]
	**Breast cancer**								
20	0	0	0	0	0	0	0	0	As for non-carriers
30	0.0008	0.0527	0.0376	0.0093	0.0012	0.0008	0.0012	0.0011	
40	0.0067	0.2327	0.1112	0.063	0.0102	0.0078	0.0109	0.0101	
50	0.0241	0.4008	0.2836	0.1934	0.0491	0.0366	0.0433	0.0399	
60	0.0597	0.5271	0.4339	0.3309	0.1016	0.092	0.091	0.084	
70	0.1016	0.6216	0.5498	0.4557	0.1565	0.1661	0.152	0.1406	
80	0.1271	0.6795	0.639	0.5336	0.1988	0.2393	0.2039	0.1891	
	**Ovarian cancer**								
Age	**Non-carrier**	***BRCA1***	***BRCA2***	***PALB2***	***CHEK2***	***ATM***	***RAD51C***	***RAD51D***	***BRIP1***
20	0.0001	0.0001	0.0001	0.0001	As for non-carriers	As for non-carriers	0.0001	0.0001	0.0001
30	0.0003	0.0007	0.0003	0.0003			0.0004	0.0003	0.0002
40	0.0007	0.0187	0.0014	0.0012			0.0014	0.0011	0.0012
50	0.0017	0.0855	0.0047	0.0041			0.0071	0.0061	0.0046
60	0.0036	0.2038	0.0679	0.0097			0.0296	0.0288	0.011
70	0.0067	0.409	0.1593	0.0184			0.0542	0.0577	0.0224
80	0.0107	0.4418	0.1784	0.03			0.064	0.079	0.0364

Abbreviations: AIHW, Australian Institute of Health and Welfare.

Table S4. Hazard ratio per standard deviation for the polygenic risk score

Cancer type	Hazard ratio per standard deviation	Source
Breast	1.65	[12]
Ovarian	1.32	[13]

The polygenic risk score (PRS) for breast cancer was based on a 313 single-nucleotide polymorphisms (SNPs). The ovarian cancer PRS was constructed from 15 SNPs.

Table S5. Tumour pathology for breast and ovarian cancer

Breast cancer		Probability
**ER positive**<br>P(ER+ ∣ BRCA1, BRCA2, PALB2, RAD51C, RAD51D, age at onset)		range 0.15-0.80
**PR positive**<br>P(PR+ ∣ ER+)		range 0.13-0.94
**HER2 overexpressing**<br>P(HER2+∣BRCA1, BRCA2, RAD51C, RAD51D, ER positive, age < 55)		range 0.03-0.33
**Grade**<br>P(High grade∣BRCA1, BRCA2, ER+, HER2+, age <55)		range 0.18-0.83
**Tumour size**<br>P(size cat ∣ BRCA1, BRCA2, detect mode, grade, ER+, HER2+, age <55)	<7mm	range 0.02-0.58
	7-9mm	range 0.04-0.28
	10-19mm	range 0.15-0.42
	20-29mm	range 0.03-0.29
	30-39mm	range 0.01-0.18
	40-49mm	range 0.002-0.11
	≥50mm	range 0.002-0.18
**Nodal status**<br>P(Node+ ∣ BRCA1, BRCA2, detect mode, tumour size, grade, ER+, age at diagnosis)	Node positive	range 0.02-0.91
**Metastatic**<br>P(Mets ∣ node+, detect mode, ER+, age at diagnosis)	Metastasis at diagnosis	range 0.007-0.14

Ovarian cancer		
**Histology**<br>P(histotype ∣ age at diagnosis, PV)	Serous	range 0.07-0.63
	Clear cell	range 0.006-0.064
	Endometrioid	range 0.02-0.24
	Mucinous	range 0.04-0.24
	Other	range 0.18-0.71
**Grade**<br>P(Grade ∣ histotype, age at diagnosis)	1	range 0.01-0.57
	2	range 0.09-0.43
	3	range 0.05-0.90
**Stage**<br>P(Stage ∣ histotype, grade)	Local	range 0.04-0.95
	Regional	range 0.17-0.38
	Distant	range 0.05-0.78

Source: [14,15]

Table S6. Breast cancer-specific mortality

Covariate	Hazard ratio (invasive)	Hazard ratio (DCIS)
Age <30	1.36	-
Age 30-39	1.18	-
Age 40-49	0.96	-
Age 60-69	0.97	-
Age 70-74	1.23	-
Age 75-79	1.84	-
Age 80-84	2.84	-
Age 85-99	3.42	-
High grade	2.77	0.27875
Size >20mm	1.35	-
Lymph node positive	1.42	-
Metastatic	9.65	-
Triple negative (year 1-5)	1.49	-
Triple negative (year 6-10)	0.58	-
HER2 positive (year 1-5)	0.68	-
HER2 positive (year 6-10)	0.51	-

Age refers to the age at breast cancer diagnosis
Source: SEER 18[14]

Table S7. Ovarian cancer-specific mortality

**Histology**	**Covariate**	**Hazard ratio**
Non-mucinous		
	Grade 1	0.45
	Grade 2	0.86
	Age <35	0.68
	Age 35-49	0.81
	Age 50-54	0.89
	Age 55-59	0.93
	Age 60-64	1.24
	Age 70-74	1.59
	Age 75-79	2.46
	Age 80-84	4.52
	Age 85-99	10.89
	Clear cell	1.28
	Endometrioid	0.72
	Other histology	2.00
	Local stage	0.11
	Regional stage	0.28
	BRCA1	0.53
	BRCA2	0.42
	BRCA1 (time-varying)	1.14
	BRCA2 (time-varying)	1.09
Mucinous		
	Grade 1	0.53
	Grade 2	0.62
	Age <25	0.73
	Age 25-34	0.49
	Age 35-49	0.81
	Age 50-54	0.89
	Age 55-59	0.95
	Age 60-64	1.29
	Age 70-74	1.18
	Age 75-79	1.76
	Age 80-84	1.61
	Age 85-99	2.11
	Local stage	0.07
	Regional stage	0.16

Age refers to the age at ovarian cancer diagnosis
Source: [14,16]

Figure S6. Assumptions related to predictive testing in relatives.

Eligibility

- Known family pathogenic variant
- No prior pathogenic variant detection
- 1 or fewer prior predictive tests
- Not an obligate non-carrier
- Not already known to carry a pathogenic variant

Obligate carriers are still eligible for predictive testing

Uptake probability for cycle

- Based on regression of uptake of cascade testing over time from Parkville FCC cohort (time zero is date of detection for family pathogenic variant)
- Probability affected by: age group, gender, degree of relation from proband, years elapsed since proband testing

Trackers

Gene detected
- Positive genetic test, known carrier

False positive
- Positive genetic test, not a carrier

True negative
- Negative genetic test, not a carrier

Obligate carrier / Obligate non-carrier
- No genetic test, carrier status based on inheritance pattern and family member testing

Predictive test
- Values 0, 1, 2 : number of predictive tests performed

Predictive eligible
- 0 or 1: eligible for a predictive test

Pathogenic variant detection
- Full pathogenic variant detection performed

Pathogenic variant detection eligible
- Eligible for a full germline pathogenic variant detection

Pathogenic variant positive outcomes

Grandparents
- Any of four grandparents:
  - Parents and piblings of other side (maternal vs paternal grandparents) are no longer eligible for predictive testing. They will not be classed as obligate non-carriers

Parents
- If one parent tests positive, other no longer eligible for predictive testing
- Piblings on other side no longer eligible for predictive testing

Piblings (aunts/uncles)
- If any test positive
  - Parent and grandparents on other side no longer eligible for predictive testing

Siblings
- No effect on any other relatives from positive test

Niblings
- Sibling (nibling parent) on proband side is an obligate carrier

Children
- No effect on any other relatives from positive test

Grandchildren
- Child (grandchild parent) is an obligate carrier

Pathogenic variant negative outcomes

Grandparents
- One side (maternal or paternal) both test negative
  - Parent (mother or father) on same side classed as obligate non-carriers and no longer eligible for predictive testing
  - Parent on other side classed as obligate carrier (but still eligible for predictive)
  - Piblings on same side no longer eligible for predictive

Parents
- Other parent is obligate carrier (still eligible for predictive)
- Piblings and grandparents on same side no longer eligible for predictive

Piblings
- No effect on other relatives from negative test

Siblings
- Niblings of sibling no longer eligible for predictive, classed as obligate non-carriers (can change if new mutation identified later)

Niblings
- No effect on other relatives from negative test

Children
- Grandchild no longer eligible for predictive, and now classed as obligate non-carriers (can change as above)

Grandchildren
- No effect on other relatives from negative test

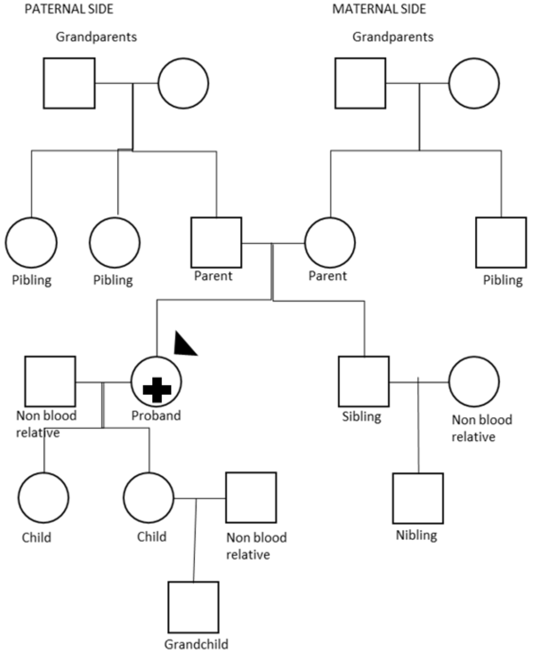

Table S8. Clinical effectiveness of risk management strategies

Risk management strategy type					Source
Relative risk	RRSO	0.19			[15]
	BRRM	0.07			
	CRRM	0.072			
Screening		Age group	Prevalent round	Subsequent rounds	
Sensitivity	Mammogram only	Age < 50	0.673	0.587	[17] Note: age <40 estimates assumed same as age 40-49
		Age 50-59	0.828	0.686	
		Age 60-69	0.896	0.769	
		Age70-79	0.865	0.824	
		Age ≥80	0.865	0.824	
	MRI only	All ages	0.887	0.772	[18]
	Combined Mammogram/MRI	All ages	0.887	0.891	
Specificity	Mammogram only	Age < 50	0.909	0.9511	[17] Note: age <40 estimates assumed same as age 40-49
		Age 50-59	0.89685	0.964967	
		Age 60-69	0.904074	0.972636	
		Age70-79	0.916597	0.972107	
		Age ≥80	0.916597	0.972107	
	MRI only	All ages	0.885	0.971	[18]
	Combined Mammogram/MRI	All ages	0.851	0.965	
Abbreviations: BRRM, bilateral risk-reducing mastectomy; CRRM, contralateral risk-reducing mastectomy; MRI, magnetic resonance imaging; RRSO, risk-reducing salpingo-oophorectomy					

Table S9. Uptake and adherence to cancer risk management strategies

Strategy				Source
**BRRM uptake**				
	BRCA1/*BRCA2*	Age-specific uptake probabilities from age 25-65[1]		15
	Non-carrier (or unknown)	No surgery		Assumption
	PALB2	Assumed same as *BRCA2* carrier		
	CHEK2	50% rate of *BRCA2*		
	Other gene	Assumed same as non-carrier		
**CRRM uptake**				
	BRCA1/*BRCA2*	0.6657 for year of diagnosis		15
	Non-carrier (or unknown)	0.047 for year of diagnosis		
	PALB2	Assumed same as *BRCA2* carrier		Assumption
	CHEK2	50% rate of *BRCA2*		
	Other gene	Assumed same as non-carrier		
**RRBSO uptake**				
	BRCA1/*BRCA2*	Age-specific uptake probabilities from age 33-75[1]		15
	Non-carrier	No surgery		Assumption
	PALB2	Assumed similar to *BRCA2* carriers from age 48		
	BRIP1	Assumed similar to *BRCA2* carriers from age 60		
	RAD51C/*RAD51D*	Assumed similar to *BRCA2* carriers from age 48		
**BreastScreen participation**			**Rate**	
	Age at first screen (years)	40-44	0.02	17
		45-49	0.03	
		50	0.09	
		51	0.09	
		52	0.10	
		53	0.12	
		54	0.13	
		55-59	0.06	
		60-64	0.009	
	Screening interval (months)	21	0.0218	Policy1-Breast microsimulation model estimates[2]
		24	0.3676	
		27	0.7404	
		30	0.8618	
		36	0.9168	
		48	0.9624	
	Moderate-risk carrier screening interval	Assumed as for population-risk, but brought forward 1 year		Assumption
**High-risk breast screening participation**				
	Age at first mammogram	*BRCA1*/*BRCA2*[1]	Median age 30.1	15
		PALB2 assumed same rates as *BRCA2*		
	Age at first MRI	*BRCA1*/*BRCA2*[1]	Median age 27.3	15
		PALB2 assumed same rates as *BRCA2*		
	Screening interval	*BRCA1*/*BRCA2*[1]		15

		PALB2 assumed same rates as *BRCA2*

[1]Uptake probabilities and screening adherence are from a previous analysis of *BRCA1* and *BRCA2* carriers seen through a Familial Cancer Service. See Petelin et al[15] for further details and estimates.

5. Model assumptions

Module	Component	Assumptions
**Input population generation**	Parity and age at birth	• The time interval between births must be less than or equal to 20 years.<br>• The time interval between births must be less than 5 years if the birthing parent has more than 6 children.<br>• The time interval between births follows a log normal distribution.
	Siblings	• Probands can have a maximum of 9 siblings.<br>• The age of the eldest sibling is relative to the proband's age within 20 years either side.<br>• The age difference between siblings follows a log normal distribution.<br>• If a proband has more than 8 siblings, the age difference must be 3 years or less.
	Partnerships	• All partners are aged within 5 years of each other.<br>• The male partner can be up to 5 years younger or 5 years older with the exception for where a woman's age at first birth was younger than 18 years; in this instance the male partner must be older.<br>• Partnerships can only occur between two individuals if they were assigned the same parity.
	Life history prior to main simulation	• Parents and grandparents must survive cancer-free until the age at their last birth.<br>• All individuals are deceased by a maximum age of 100.<br>• A maximum of three events can occur prior to entry into the main simulation, including: (i) breast cancer, (ii) ovarian cancer, (iii) breast cancer death, (iv) ovarian cancer death, or (v) other-cause death.
**Cancer natural history**	• The same distribution of histotype was used in women aged 70 and over at diagnosis as for women aged 50-69; while the distribution was different for women in the SEER cohort, based on expert advice many ovarian cancers classified as other histology in women are often serous histology.<br>• A second primary breast cancer can only occur within the first 20 years after a diagnosis of the first primary breast cancer.	
**Mortality**	• All individuals are deceased by age 100 years.	

	• Women diagnosed with breast and/or ovarian cancer are at risk of cancer-specific death for a maximum of ten years following diagnosis.
**Genetic testing**	• Genetic referral and testing triggered by a cancer diagnosis must occur in the same year as diagnosis.<br>• See specific genetic testing assumptions regarding predictive testing in Figure S6.
**Cancer risk management**	• Women attending for breast MRI are assumed to be participating in screening through a specialised high-risk management clinic.<br>• No risk-reducing surgery or high-risk breast cancer screening occurs outside confirmed P/LP variant carriers. That is, women with a strong family history are only eligible for standard population-based mammographic screening.

Note: Further assumptions regarding cancer risk management and mortality can be found in Petelin et al. 2018.[15]

6. Population validation

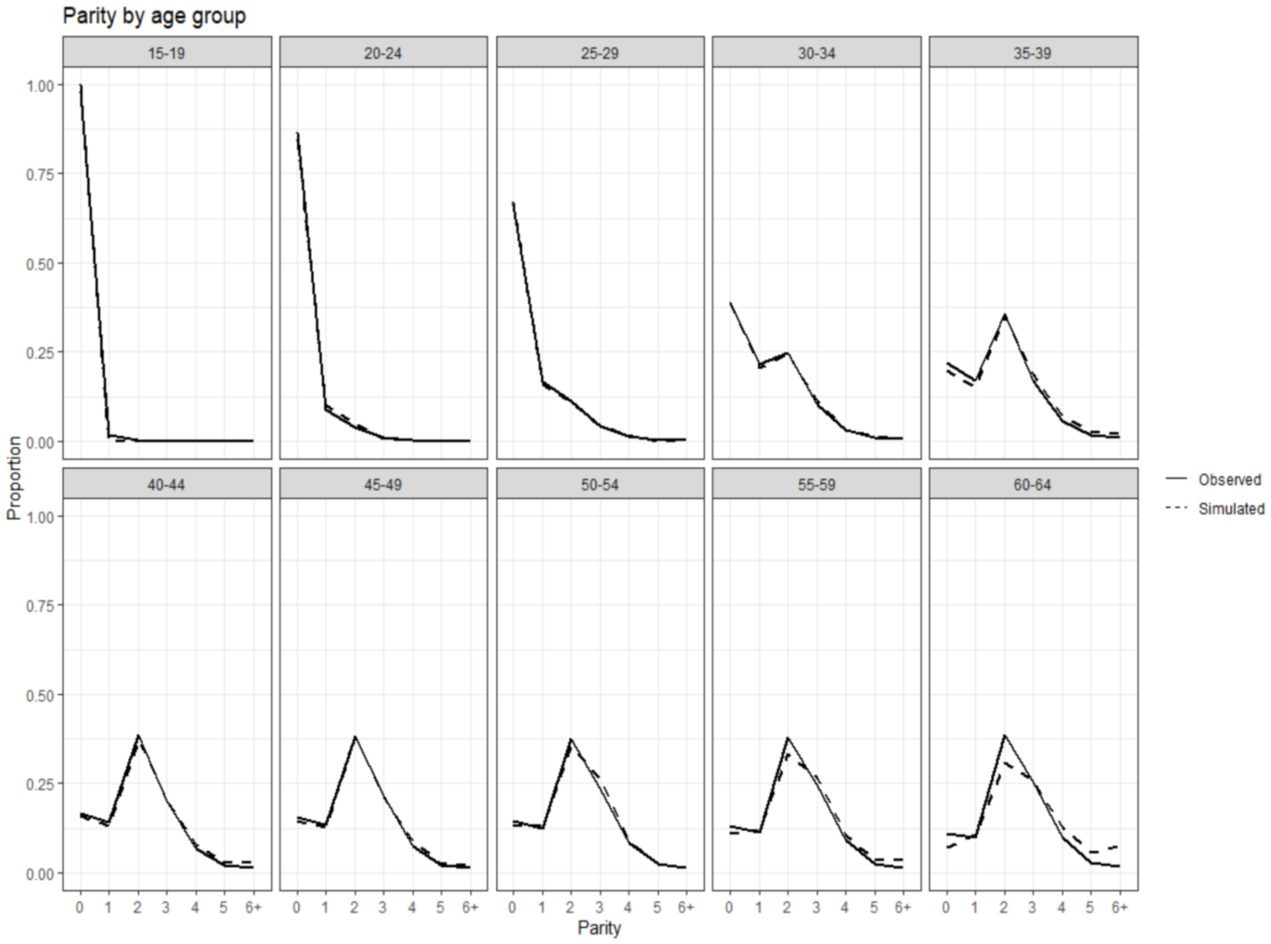

Figure S7. Parity by age group in observed versus simulated population. Simulated data were compared to observed data published by the Australian Bureau of Statistics.[19]

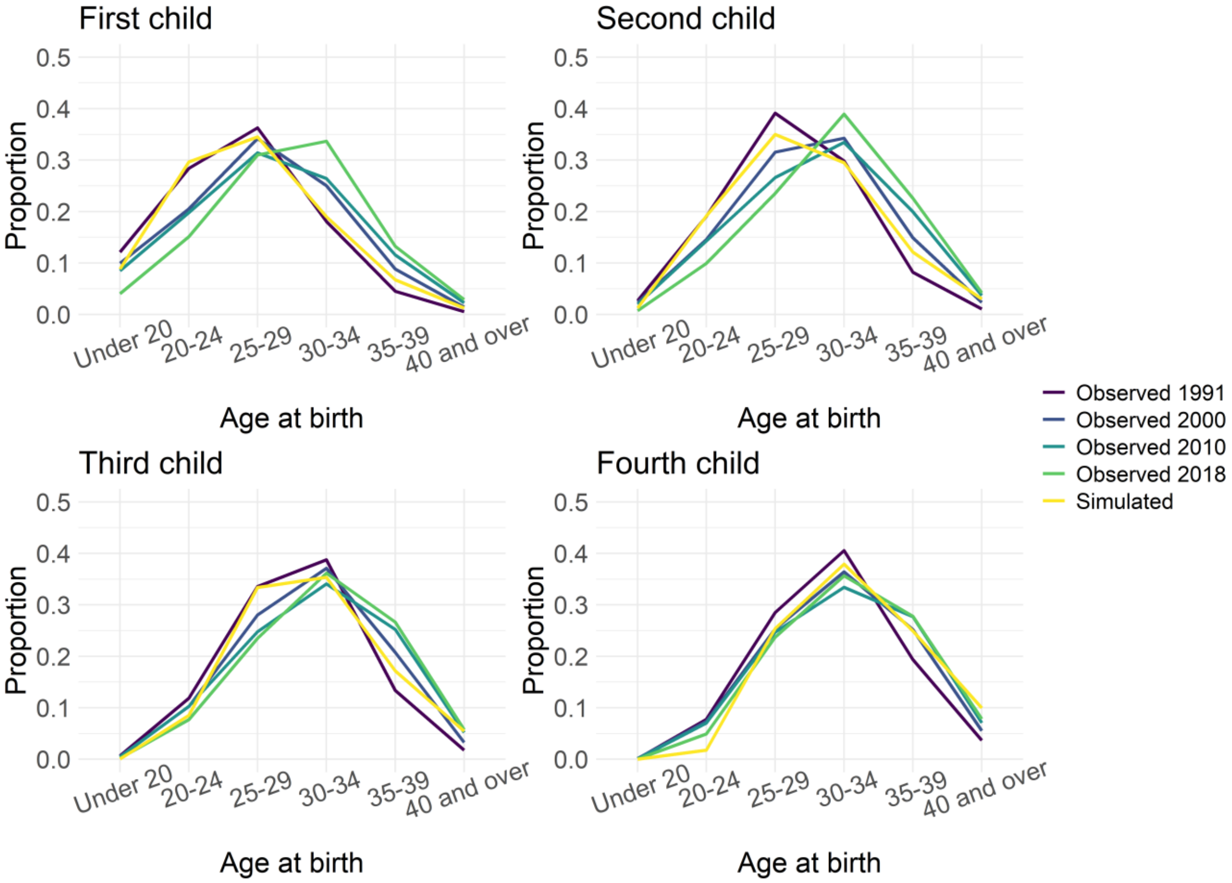

Figure S8. Distribution of birth ages by child order compared to observed Australian population data.

Simulated data were compared to observed data published by the Australian Bureau of Statistics.[19]

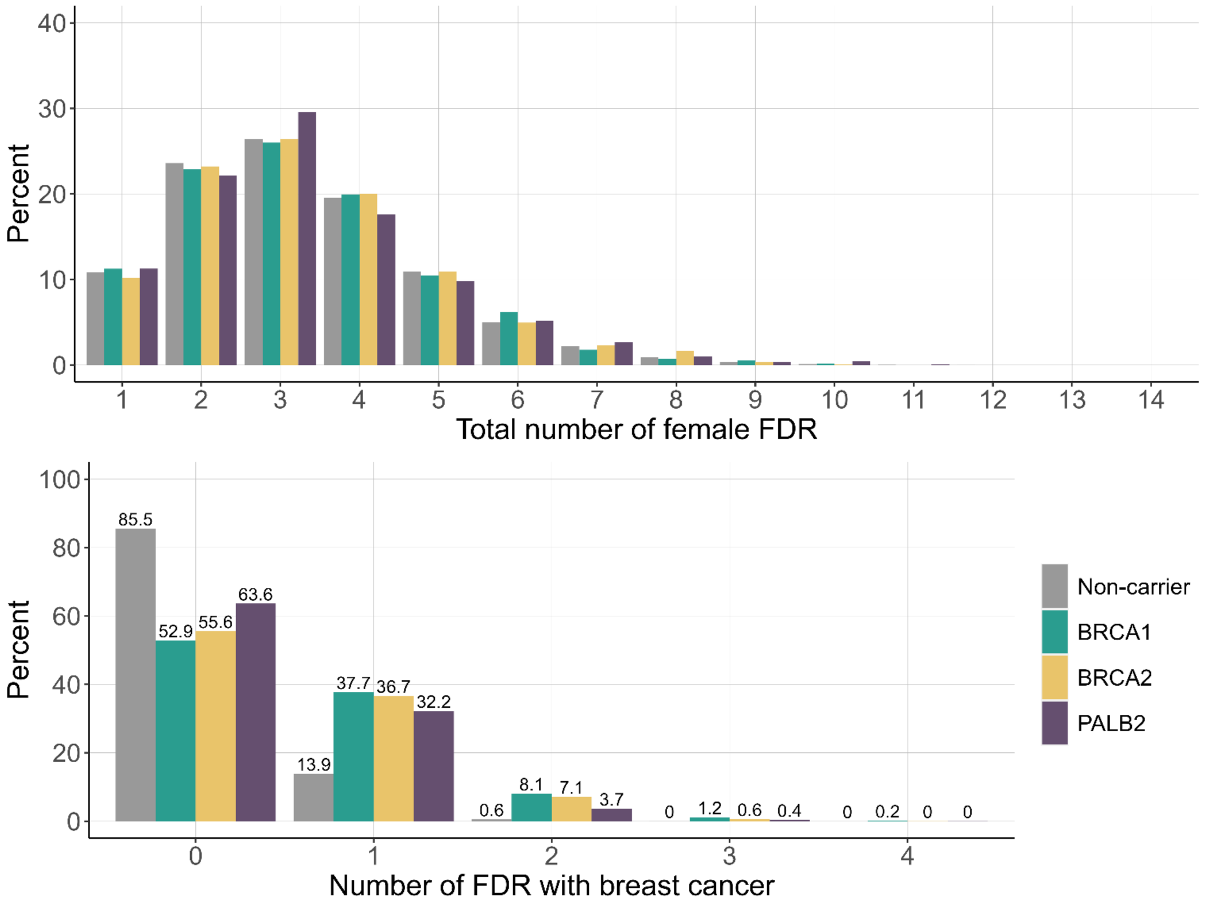

Figure S9. Family history of breast cancer in first degree relatives by gene. Abbreviations: FDR, first degree relatives.

Table S10. Validation of prevalence of pathogenic variants by personal history of cancer.

Simulated input population		Simulated Mean (95% CI)	Observed Mean (95% CI)	Source
**Personal history of triple negative breast cancer**				
Women only	*ATM*	0.0012 (0-0.0204)		
mean age 63.5	*BRCA1*	0.0486 (0.0311-0.0678)	0.0592 (0.0364-0.0862)	
range (22-98)	*BRCA2*	0.0174 (0-0.0366)	0.0342 (0.0114-0.0611)	
	BRIP1	0.0012 (0-0.0204)		
	CHEK2	0.0137 (0-0.0329)		[20]
	PALB2	0.0075 (0-0.0267)		
	RAD51C	0.0012 (0-0.0204)		
	RAD51D	0.0025 (0-0.0217)		
	non-carrier	0.9066 (0.8892-0.9258)	0.9066 (0.8838-0.9336)	
**Personal history of non-mucinous ovarian cancer**				
Women only	*ATM*	0.0006 (0-0.0130)		
mean age 64.77	*BRCA1*	0.0699 (0.0578-0.0822)	0.0879 (0.0679-0.1087)	
range 18-80	*BRCA2*	0.0617 (0.0497-0.0741)	0.0529 (0.033-0.0737)	
	BRIP1	0.0042 (0-0.0166)	0.0104	
	CHEK2	0.0078 (0-0.0202)	0.0036	*BRCA1/2*[21] Non-*BRCA1/2*[5]
	PALB2	0.0081 (0-0.0205)	0.0047	
	RAD51C	0.0039 (0-0.0163)	0.0037	
	RAD51D	0.0026 (0-0.0150)	0.0042	
	non-carrier	0.8411 (0.8291-0.8534)	0.835	

7. Cancer outcomes validation

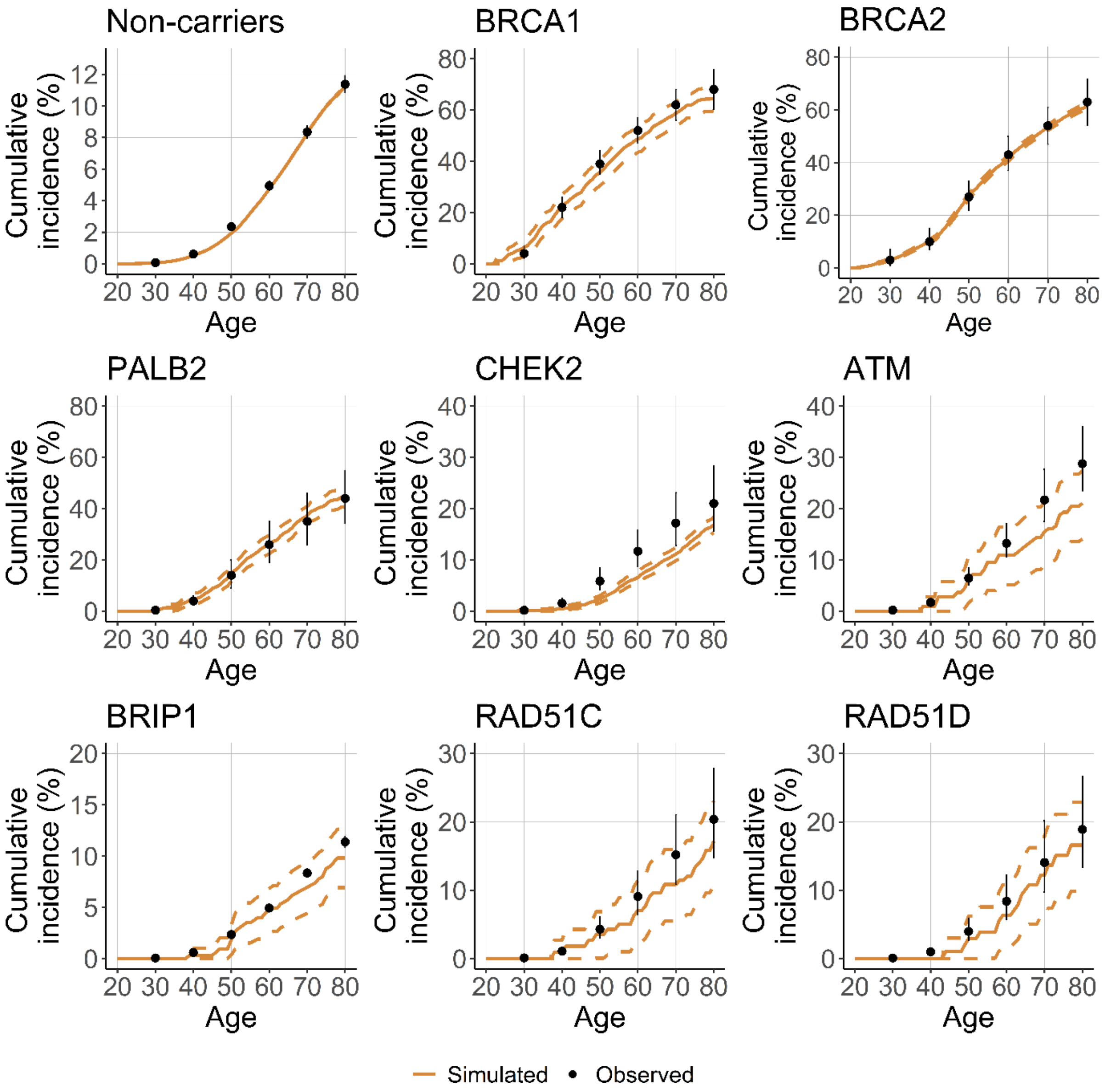

Figure S10. Cumulative breast cancer incidence by gene.
Simulated outcomes were validated against the original input data (internal validation).

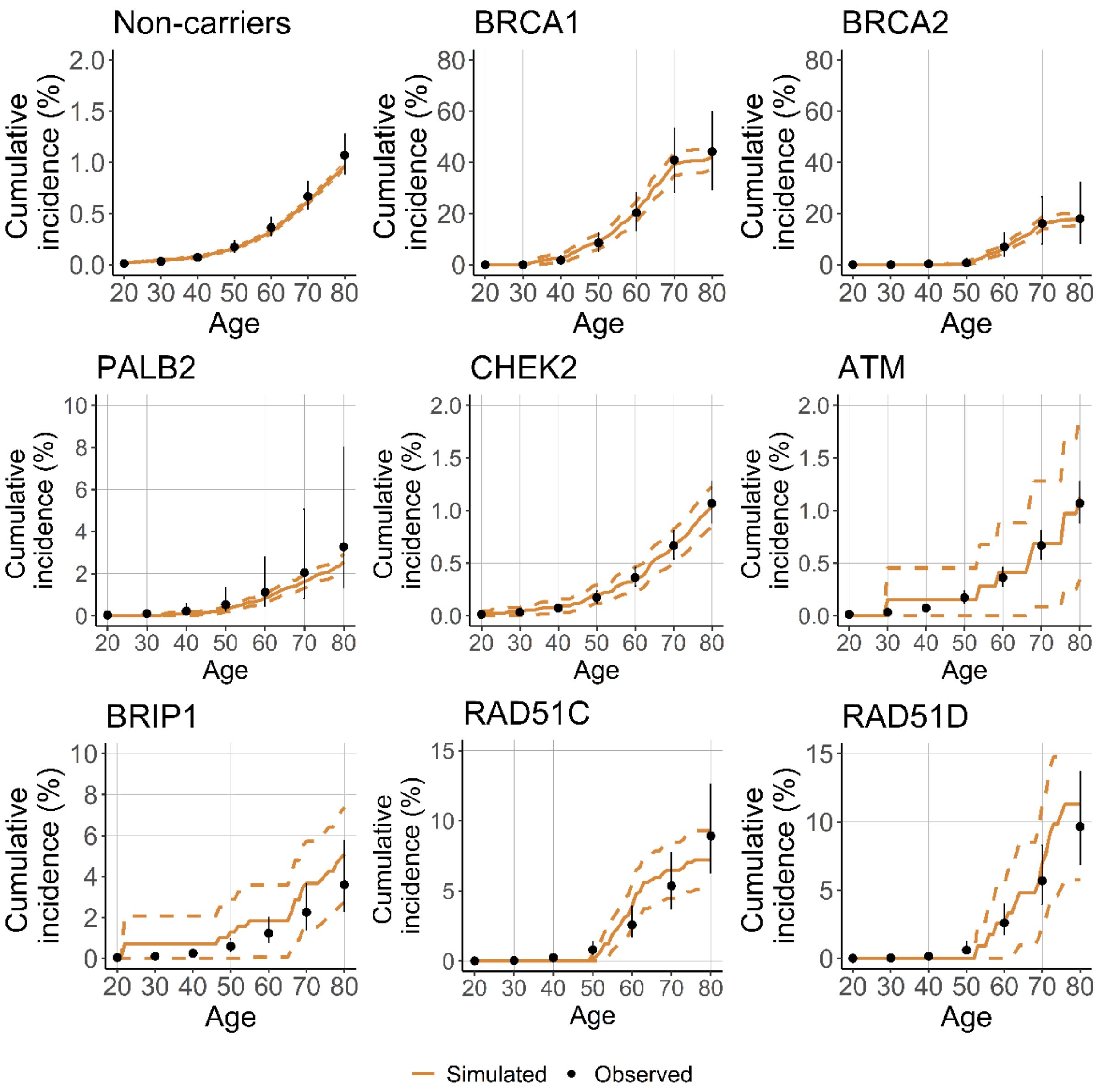

Figure S11. **Cumulative ovarian cancer incidence by gene.**
Simulated outcomes were validated against the original input data (internal validation).

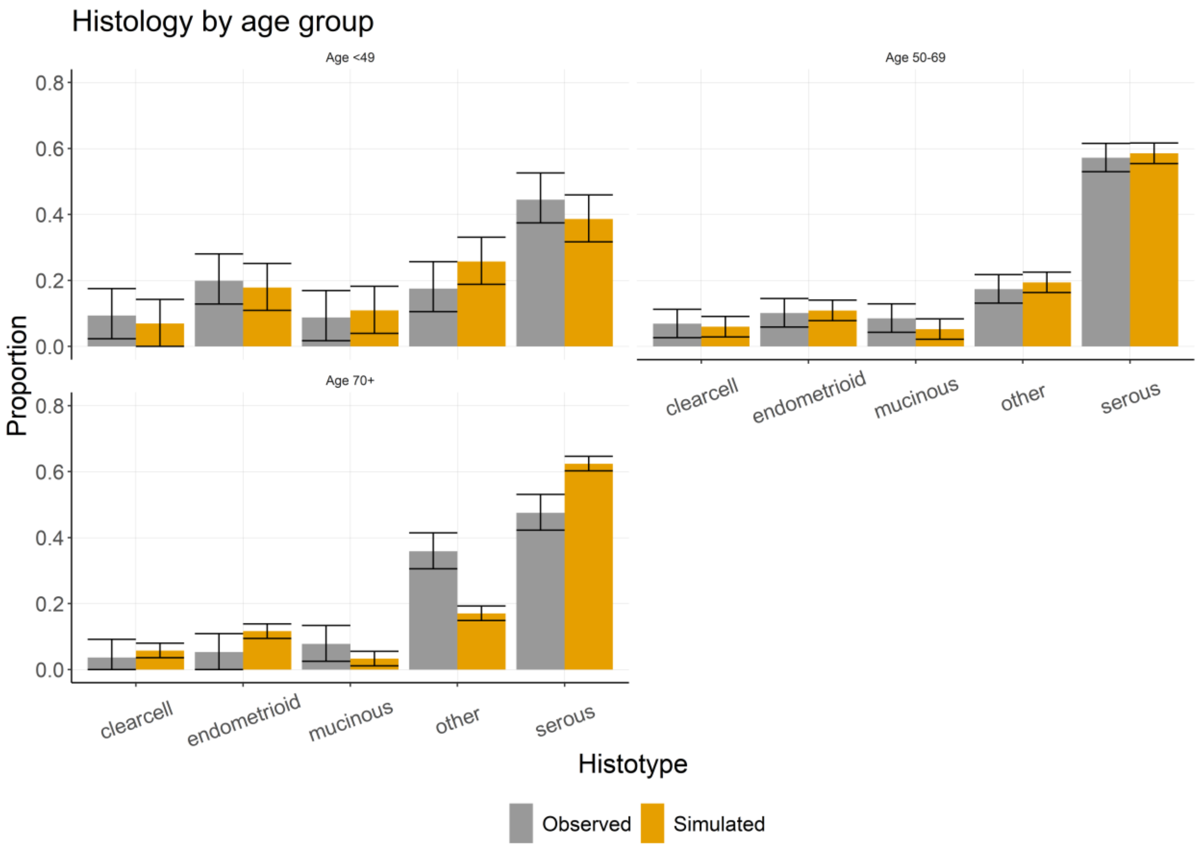

Figure S12. **Distribution of ovarian cancer histology by age group.** Simulated outcomes were compared to the observed distribution from the Australian Institute of Health and Welfare.[22]

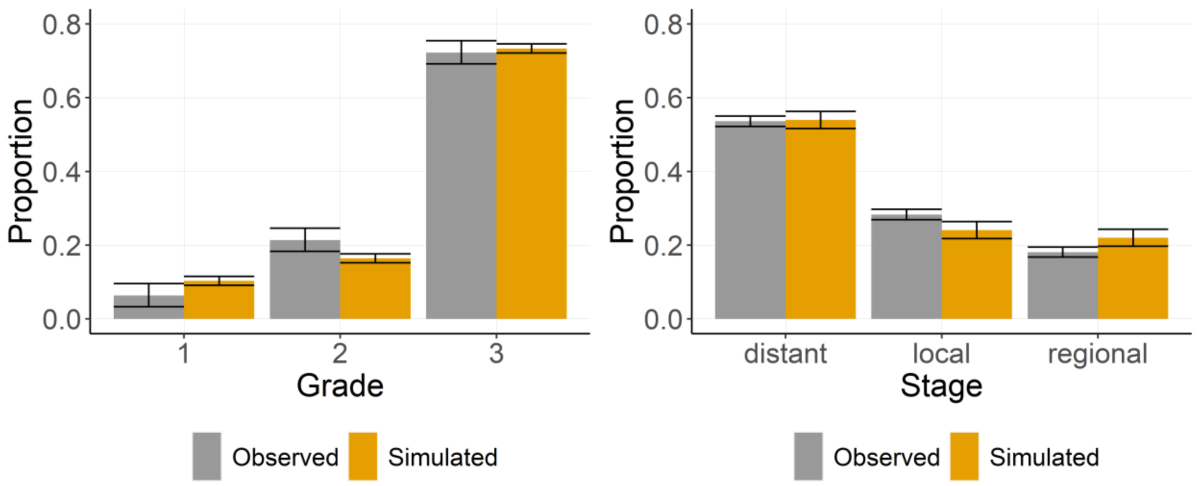

Figure S13. **Distribution of ovarian cancer grade and stage** Simulated outcomes were compared using published data from the Australian Ovarian Cancer Study.[21]

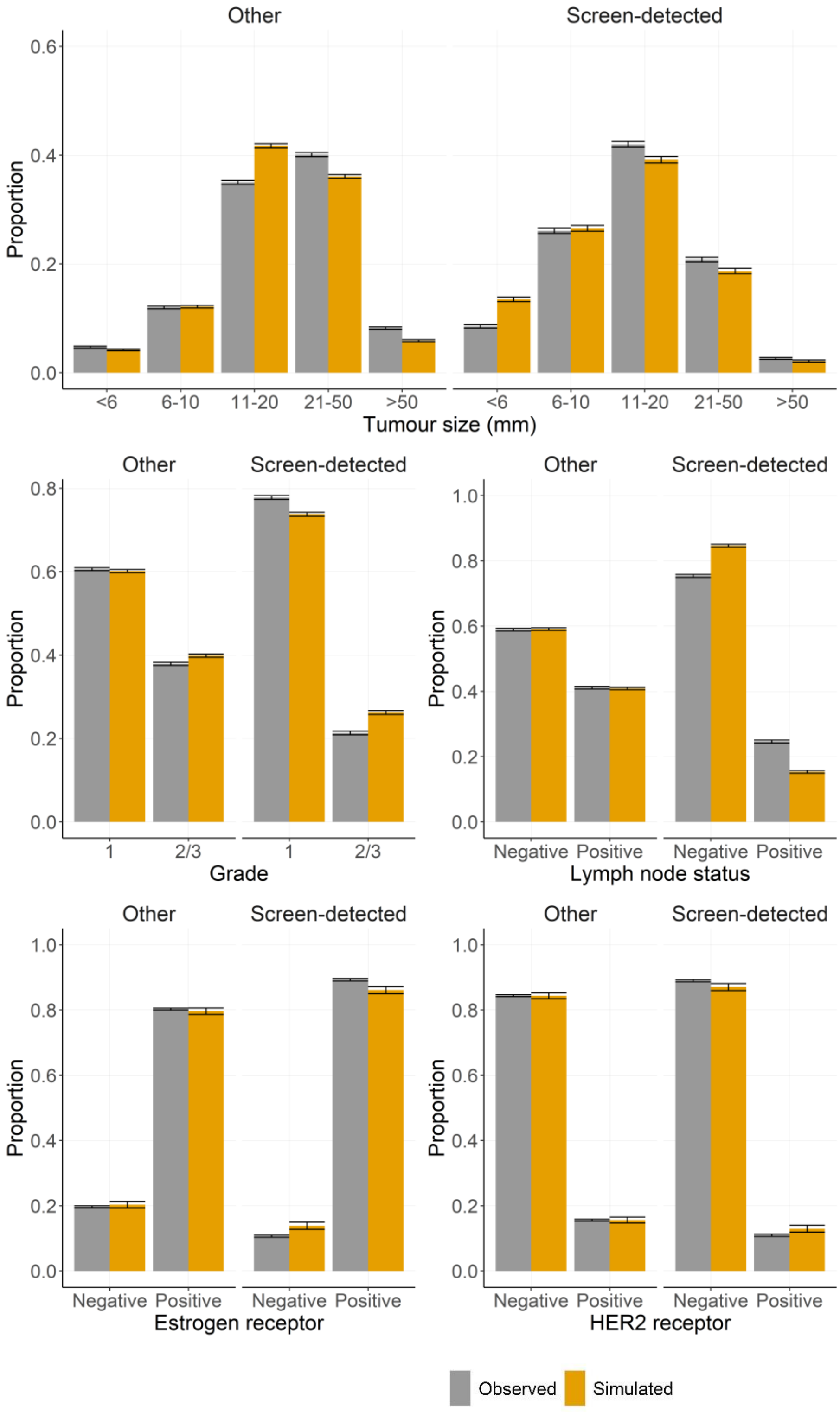

Figure S14. **Distribution of breast cancer pathology by mode of detection**
Simulated outcomes by mode of detection were compared to a cohort of breast cancer cases diagnosed in Australi.[23]

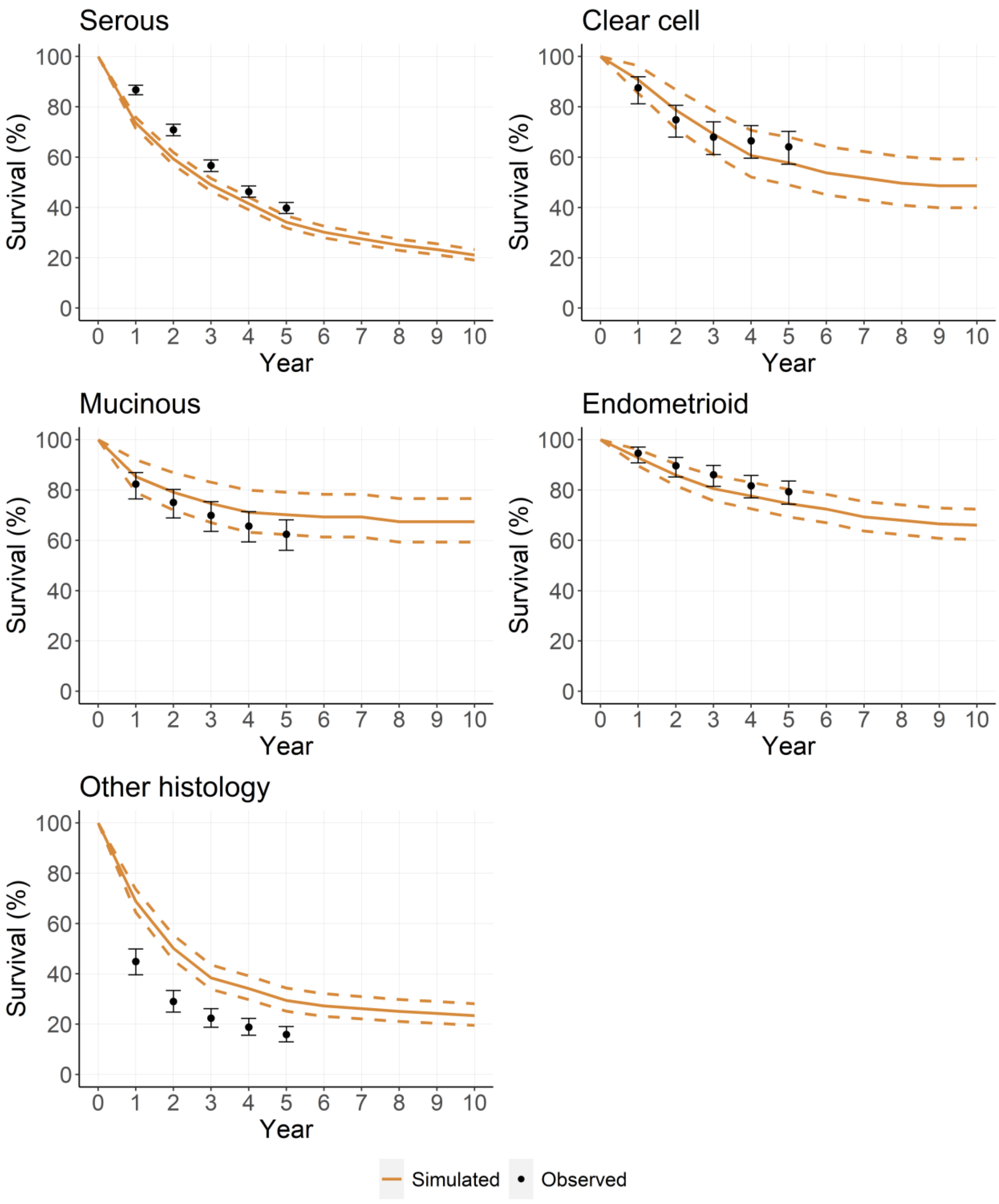

Figure S15. **Relative survival after ovarian cancer by histology.** Simulated output was validated using observed ovarian cancer mortality in Australia from the Australian Cancer Database.[24]

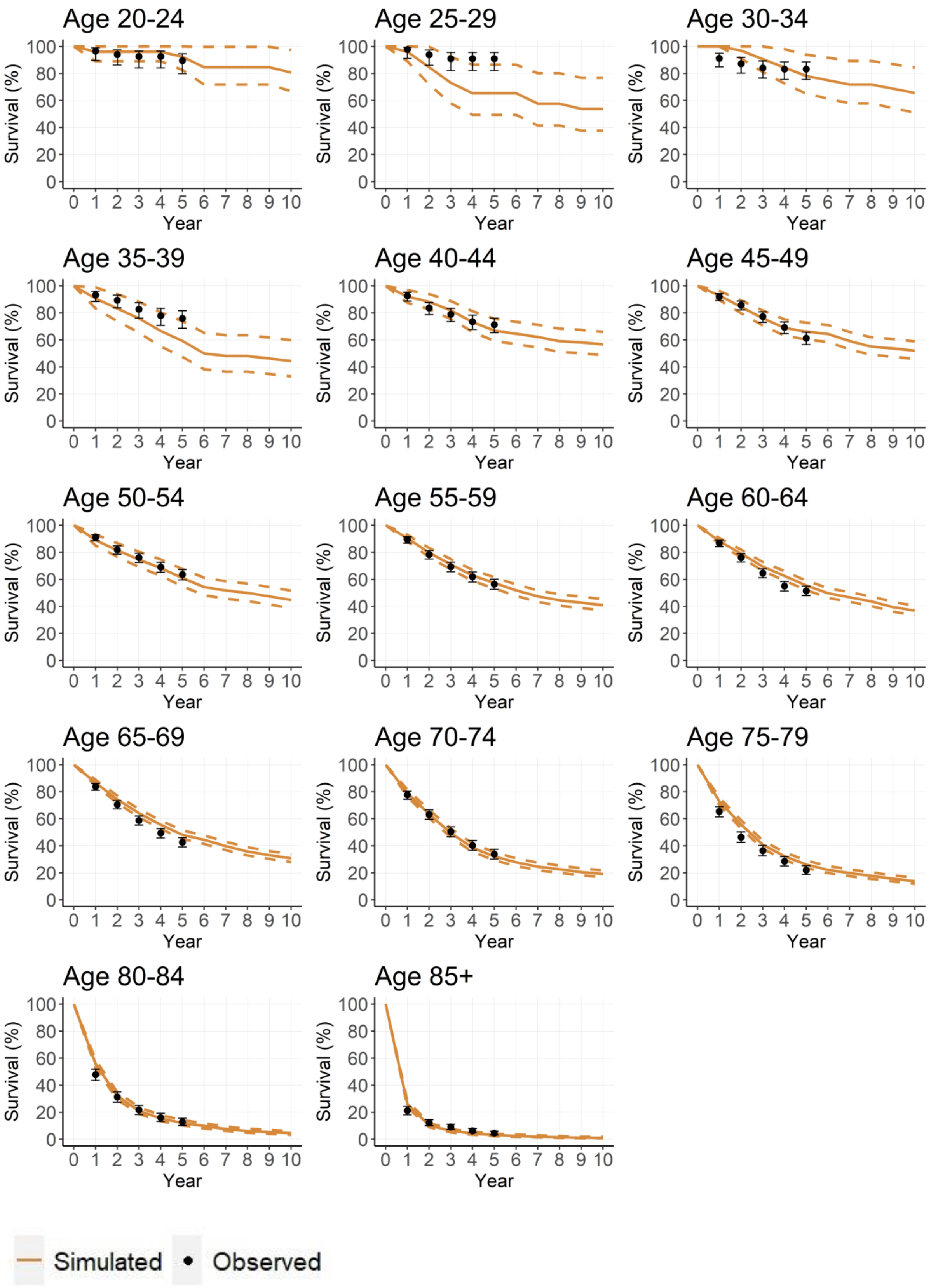

Figure S16. **Relative survival after ovarian cancer by age group.**
Simulated output was validated using observed ovarian cancer mortality in Australia from the Australian Cancer Database.[24]

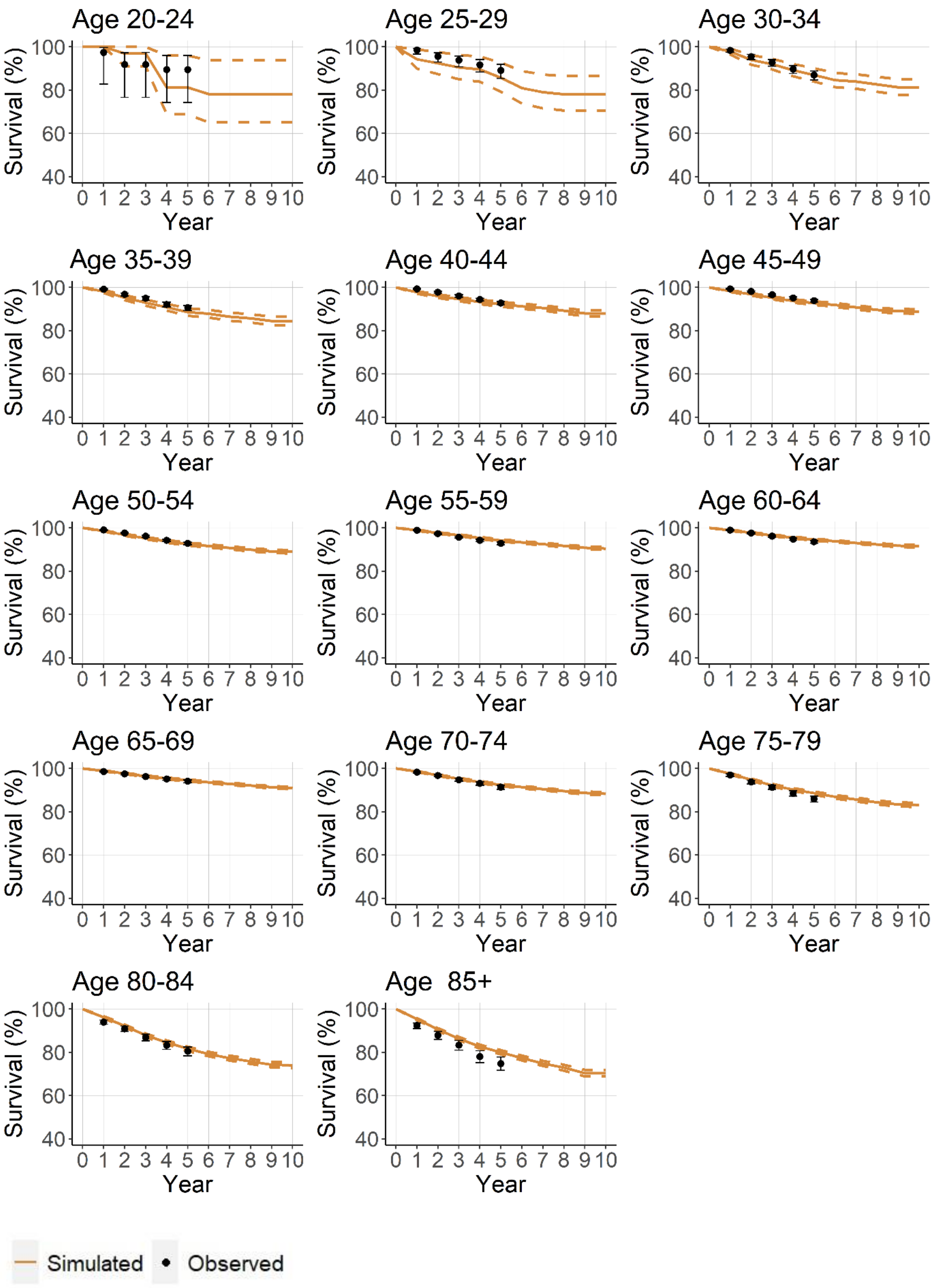

Figure S17. Relative survival after breast cancer by age.
Simulated output was validated using observed ovarian cancer mortality in Australia from the Australian Cancer Database.[24]

8. Genetic testing validation

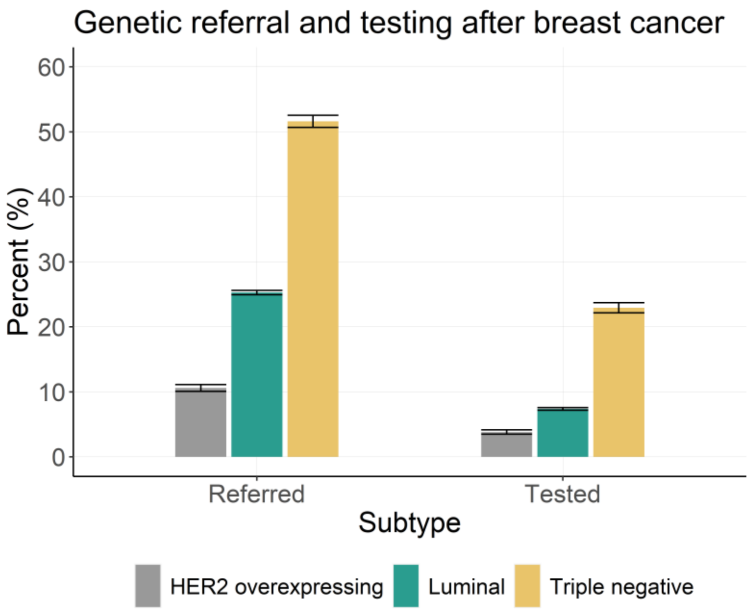

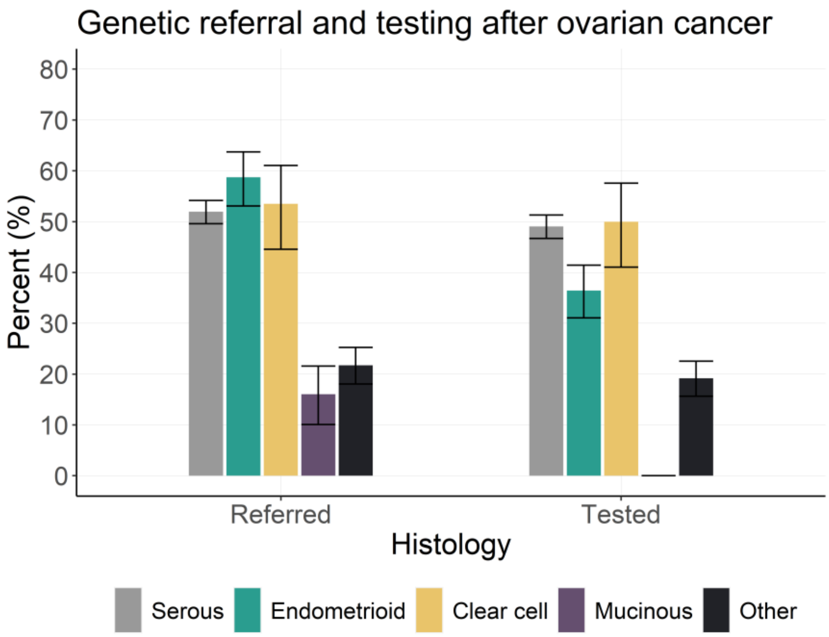

Figure S18. **Simulated referral rates for genetic counselling and genetic testing after a cancer diagnosis.**

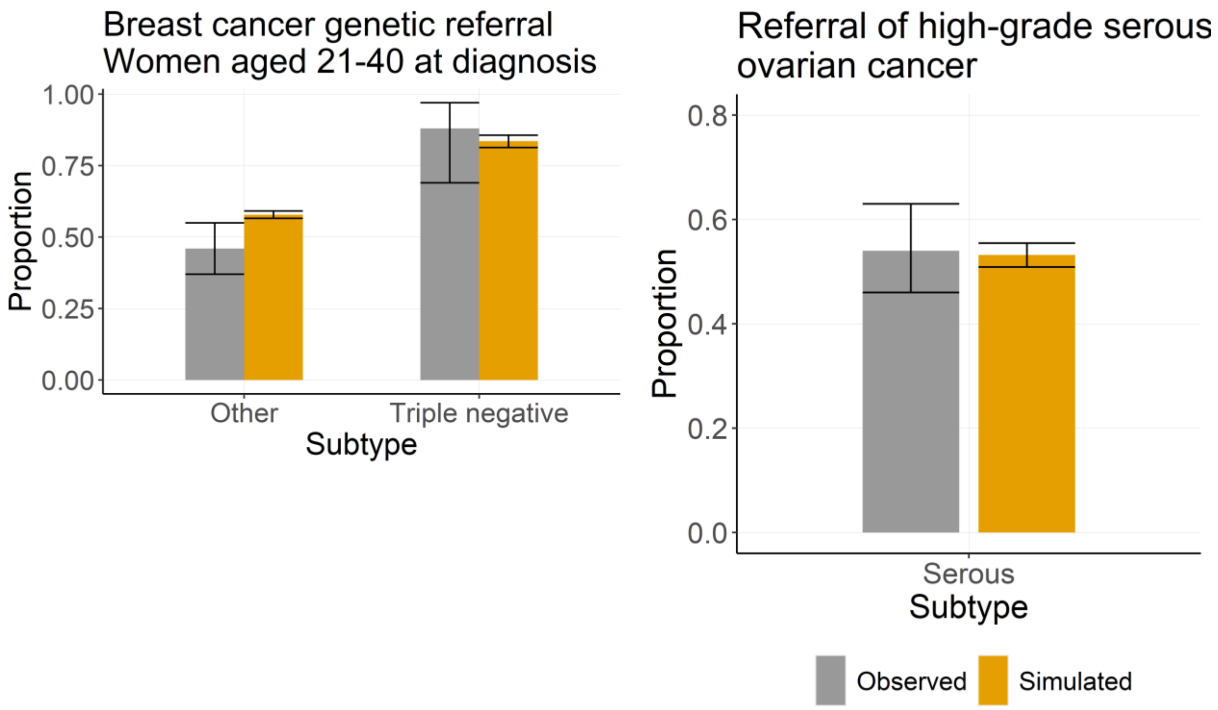

Figure S19. Validation of referral rates for young-onset breast cancer and high-grade serous ovarian cancer.

Simulated output was validated using published genetic testing referral rates.[25,26]

Predictive testing in all relatives

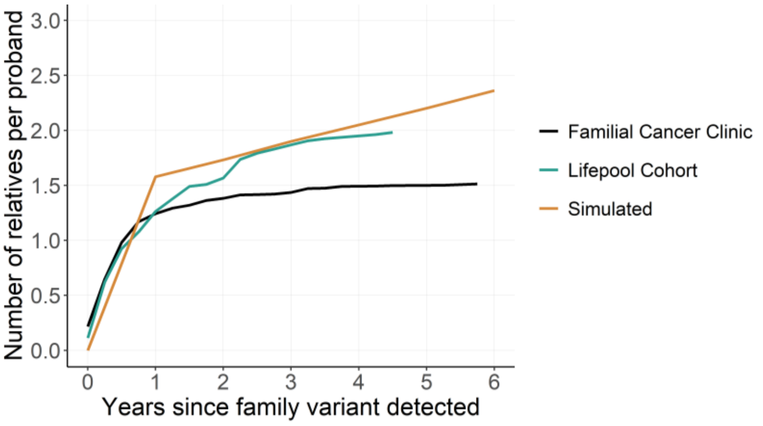

Predictive testing by gender

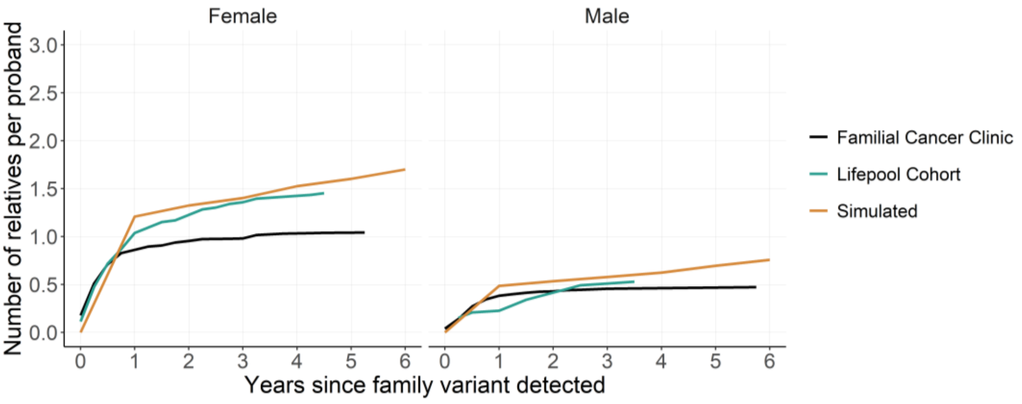

Predictive testing by degree of relation

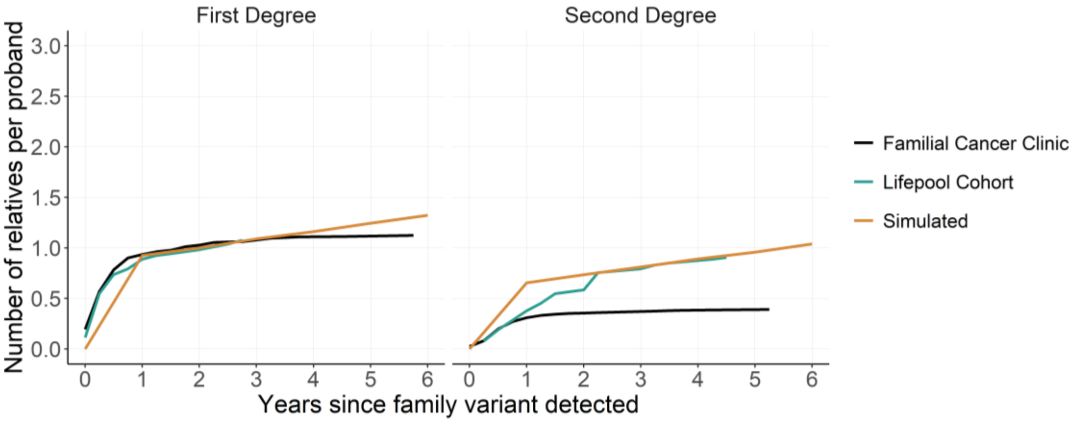

Figure S20. Uptake of predictive testing in relatives compared to observed data. Simulated output was compared to genetic testing uptake by relatives recorded in the Parkville Familial Cancer Clinic database over the period 1996-2016, and to uptake by relatives of participants in the Lifepool study (population-based, unpublished observations).

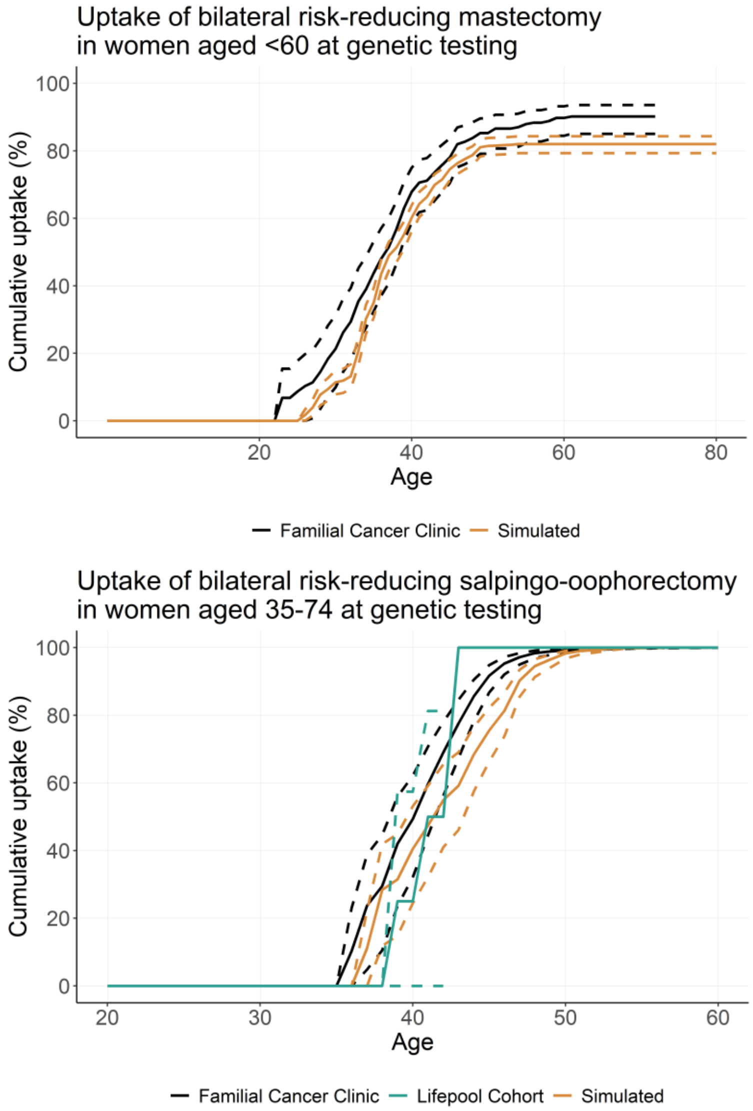

Figure S21. Validation of risk-reducing surgery uptake in *BRCA1*/2 pathogenic/likely pathogenic variant carriers

Kaplan-Meier survival curves for age at risk-reducing surgery. Women were included if they were unaffected by cancer at the time of genetic testing. Entry was at the age of genetic testing. For bilateral risk-reducing mastectomy, women were censored at age at breast or ovarian cancer diagnosis, age at death, or age at last follow-up (or simulation exit for modelled women). Women were censored at ovarian cancer diagnosis, death, or last known follow-up for bilateral risk-reducing salpingo-oophorectomy uptake. The validation cohorts were women from the Parkville Familial Cancer Centre who were tested between 1996-2016, and women identified as pathogenic/likely pathogenic variant carriers through the Lifepool study (unpublished observations).

9. Model outcomes

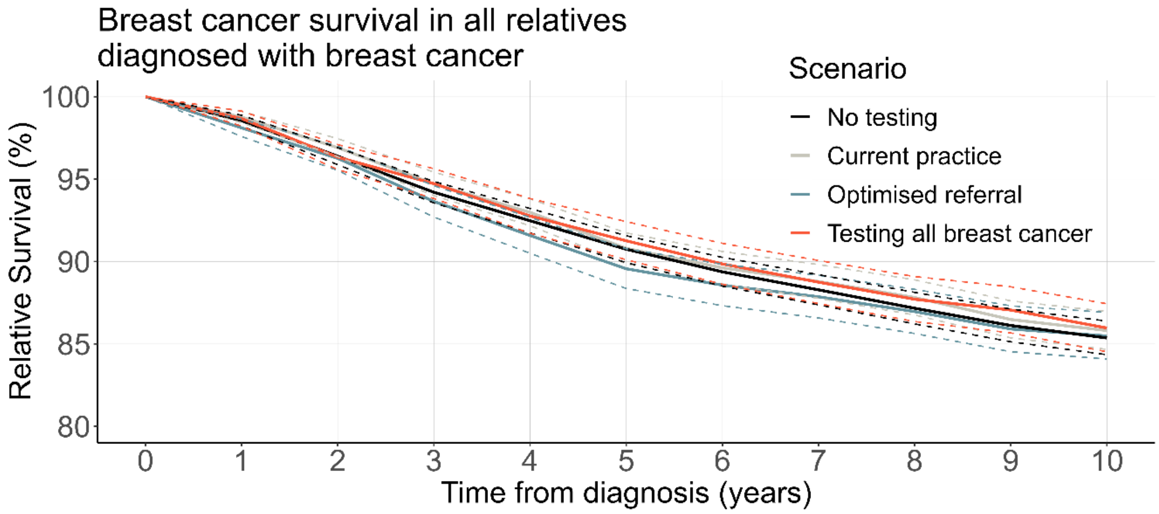

Number at risk

Scenario	0	1	2	3	4	5	6	7	8	9	10
No testing	5087	4889	4672	4469	4282	4100	3928	3795	3662	3522	3404
Current practice	3889	3771	3614	3461	3321	3146	3023	2927	2827	2715	2615
Optimised referral	2591	2495	2391	2269	2176	2075	2005	1954	1895	1825	1766
Testing all breast cancer	2408	2321	2216	2124	2035	1948	1868	1801	1735	1679	1612

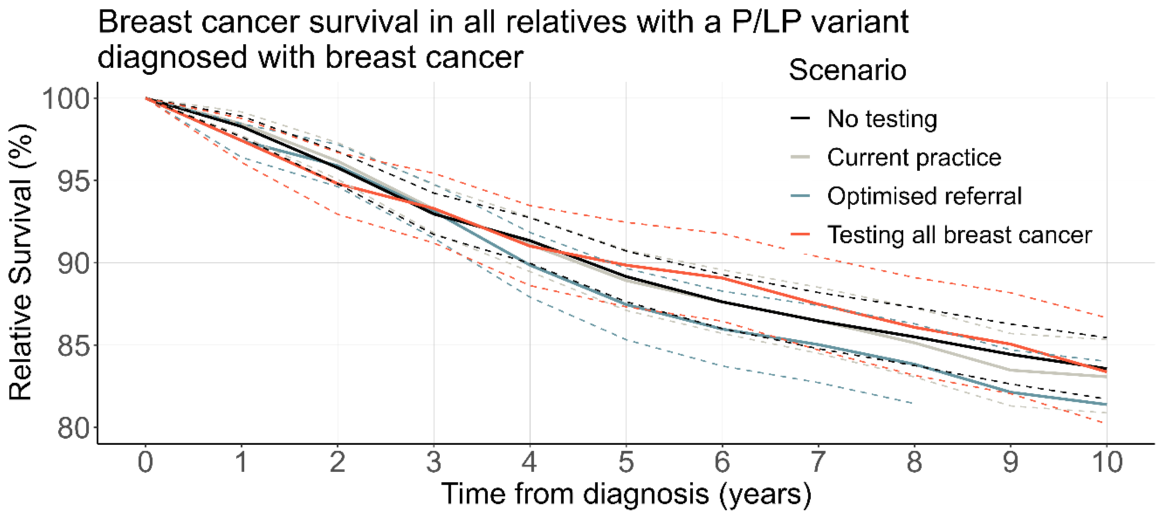

Number at risk

Scenario	0	1	2	3	4	5	6	7	8	9	10
No testing	1620	1569	1502	1435	1382	1335	1287	1249	1206	1166	1125
Current practice	1153	1128	1088	1040	1002	956	923	901	872	839	810
Optimised referral	929	895	864	827	790	761	730	712	690	661	639
Testing all breast cancer	543	520	504	490	472	460	448	436	422	404	386

Figure S22. Breast cancer survival after a cancer diagnosis in relatives of pathogenic/likely pathogenic variant carriers.

Relatives included only women related to *BRCA1*/*BRCA2*/*PALB2* pathogenic/likely pathogenic variant carriers, and who were unaffected by cancer at the time of the proband's breast cancer diagnosis.

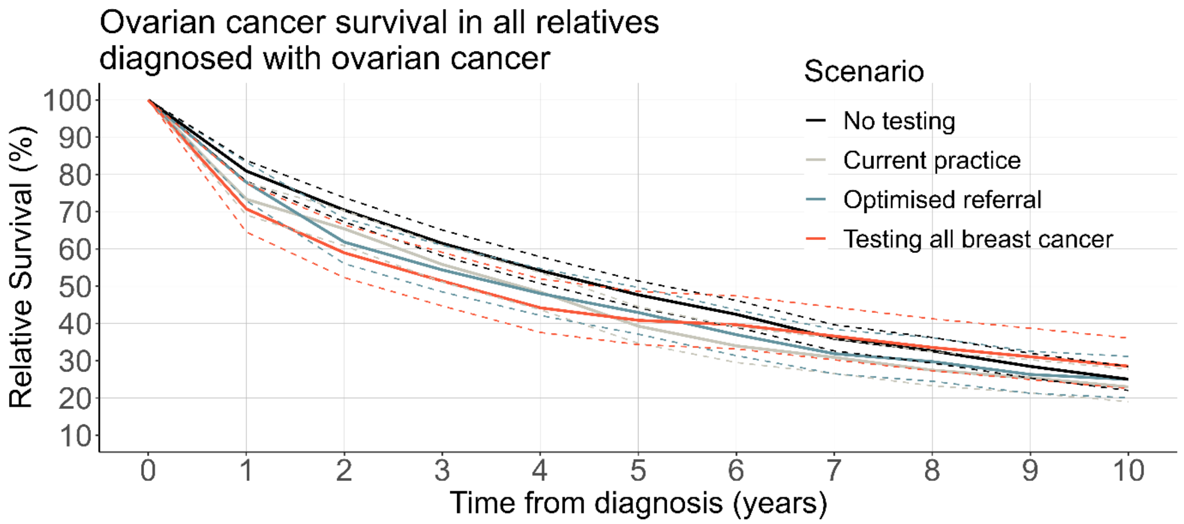

Number at risk

Scenario	0	1	2	3	4	5	6	7	8	9	10
No testing	765	610	520	446	391	337	295	248	220	190	161
Current practice	394	286	253	211	180	141	121	108	93	84	76
Optimised referral	249	189	149	129	113	101	87	75	69	60	56
Testing all breast cancer	188	132	109	93	79	69	65	60	54	49	45

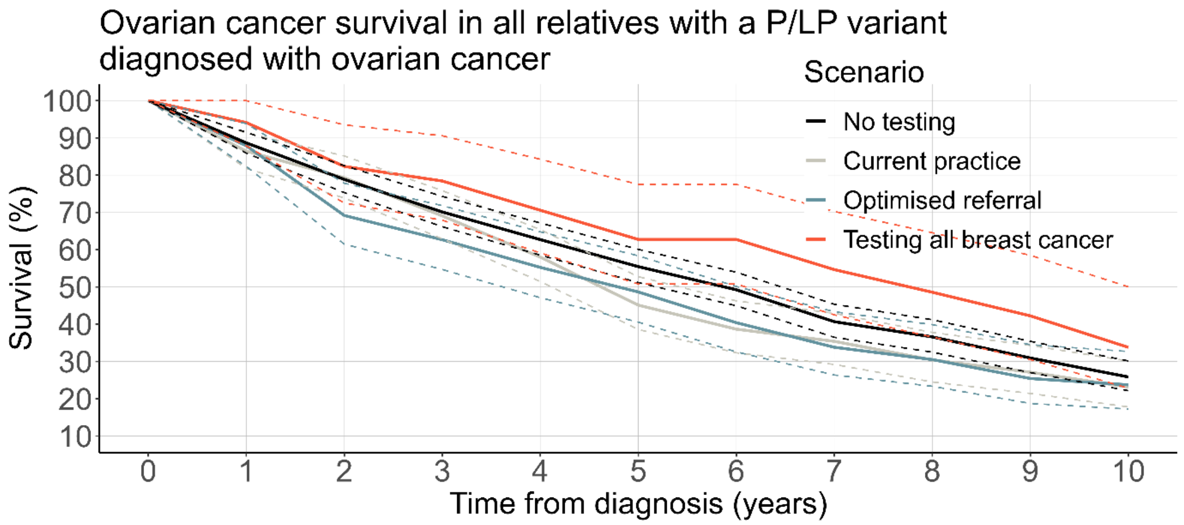

Number at risk

Scenario	0	1	2	3	4	5	6	7	8	9	10
No testing	494	434	379	331	294	259	230	189	168	140	114
Current practice	199	170	155	131	108	84	71	64	55	48	41
Optimised referral	124	108	85	76	67	59	49	41	36	30	27
Testing all breast cancer	51	48	42	40	36	31	31	27	23	20	16

Figure S23. Ovarian cancer survival after a cancer diagnosis in relatives of pathogenic/likely pathogenic variant carriers.

Relatives included only women related to *BRCA1*/*BRCA2*/*PALB2* pathogenic/likely pathogenic variant carriers, and who were unaffected by cancer at the time of the proband's breast cancer diagnosis.

Table S11. Genetic outcomes in the relatives of probands diagnosed with breast cancer, including age groups

First- and second-degree relatives of probands who have a P/LP variant		Scenario 1: No genetic testing		Scenario 2: Current practice		Scenario 3: Optimised referral of breast and ovarian cancer		Scenario 4: Genetic testing for all breast cancers	
Number of full sequencing genetic tests, n (%) [1]		0 (0%)		1438 (4.88%)		2238 (8.04%)		3938 (17.71%)	
Total number of female relatives, n[2]		34 169		29 482		27 815		22 237	
Number of predictive tests (male and female relatives), n (%)		0 (0%)		10 813 (19.08%)		13 705 (25.37%)		19 621 (45.26%)	
Total number of male and female relatives, n		66 358		56 672		54 020		43 352	
	**Age group**	**N**	**Average[3]**	**N**	**Average[3]**	**N**	**Average[3]**	**N**	**Average[3]**
Number of female relatives of proband P/LP variant carriers, unaffected by cancer at the time of proband diagnosis	Under 18	7633	1.80	6565	1.79	6557	1.85	5164	1.80
	Age 18-29	5997	1.41	5191	1.41	4685	1.32	3778	1.32
	Age 30-39	3896	0.92	3236	0.88	3044	0.86	2561	0.89
	Age 40-49	3352	0.79	2860	0.78	2743	0.77	2014	0.70
	Age 50-59	2638	0.62	2236	0.61	1981	0.56	1811	0.63
	Age 60-69	2495	0.59	2307	0.63	2234	0.63	1822	0.64
	Age 70-89	4326	1.02	3937	1.07	3761	1.06	3094	1.08
	Age ≥90	399	0.09	329	0.09	314	0.09	217	0.08
	**Total**	**30 736**	**7.24**	**26 661**	**7.26**	**25 319**	**7.14**	**20 461**	**7.14**
	**Median age (IQR)**	34 (18, 58)		34 (18, 59)		34 (17, 59)		34 (17, 60)	
	**Age group**	**N**	**Average[3]**	**N**	**Average[3]**	**N**	**Average[3]**	**N**	**Average[3]**
Number of female relatives with a P/LP variant, unaffected by cancer at the time of proband diagnosis	Under 18	2251	0.53	1939	0.53	1899	0.54	1497	0.52
	Age 18-29	1964	0.46	1723	0.47	1519	0.43	1269	0.44
	Age 30-39	1377	0.32	1081	0.29	1047	0.30	884	0.31
	Age 40-49	1167	0.27	1003	0.27	941	0.27	670	0.23
	Age 50-59	801	0.19	634	0.17	588	0.17	557	0.19
	Age 60-69	550	0.13	532	0.14	519	0.15	444	0.15
	Age 70-89	715	0.17	685	0.19	660	0.19	531	0.19

	Age ≥90	56	0.01	36	0.01	44	0.01	29	0.01
	**Total**	**8881**	**2.08**	**7633**	**2.07**	**7217**	**2.06**	**5881**	**2.04**
	**Median (IQR)**	31 (17, 48)		31 (17, 49)		31 (17, 50)		31 (17, 51)	
	**Gene**	**N**	**%**	**N**	**%**	**N**	**%**	**N**	**%**
Prevalence of P/LP variants in unaffected female relatives	*BRCA1*	1841	20.73	1504	19.70	1284	17.79	1263	21.48
	BRCA2	3955	44.53	3310	43.36	3065	42.47	2477	42.12
	PALB2	3085	34.74	2819	36.93	2868	39.74	2141	36.41
	**Total**	**8881**	**100**	**7633**	**100**	**7217**	**100**	**5881**	**100**
P/LP variant detected during lifetime[4]	*BRCA1*	0	0	1109	73.74	1103	85.9	1149	90.97
	BRCA2	0	0	1477	44.62	1905	62.15	2147	86.68
	PALB2	0	0	594	21.07	965	33.65	1710	79.87
	**Total**	**0**	**0**	**3180**	**41.66**	**3973**	**55.05**	**5006**	**85.12**
	**Age group**			**Diagnostic sequence**	**Predictive test**	**Diagnostic sequence**	**Predictive test**	**Diagnostic sequence**	**Predictive test**
Method of gene detection	Under 18	**-**	-	25.82	74.18	29.27	70.73	28.12	71.88
	Age 18-29	**-**	-	29.86	70.14	29.50	70.50	15.35	84.65
	Age 30-39	**-**	-	26.88	73.12	29.02	70.98	14.01	85.99
	Age 40-49	**-**	-	25.85	74.15	28.43	71.57	19.87	80.13
	Age 50-59	**-**	-	26.24	73.76	36.84	63.16	35.46	64.54
	Age 60-69	**-**	-	21.77	78.23	37.65	62.35	40.41	59.59
	Age 70-89	**-**	-	15.15	84.85	30.10	69.90	24.00	76.00
	Age ≥90	**-**	-	0.00	100.00	20.00	80.00	11.11	88.89
	**All ages**	**-**	**-**	26.73	73.27	30.05	69.95	22.81	77.19

[1]Female relatives only, including those already affected by breast and/or ovarian cancer at the time of the proband's diagnosis. These include full sequence genetic tests and predictive tests unrelated to proband genetic testing or proband breast cancer diagnosis (e.g. another non-proband relative is found to carry a P/LP variant). For example, of the 1438 full sequence detections under the current practice scenario, 1181 (82%) were women unaffected at the time of the proband breast cancer diagnosis but who were later diagnosed with cancer, with the remainder being those tested after a personal cancer diagnosis prior to the proband's diagnosis.

[2]Includes female relatives already affected by cancer at the time of the proband's diagnosis.

[3]Averages are per proband with a P/LP variant. For example, for current practice there were 3670 proband P/LP variant carriers (see Table 3), and a total of 26 661 female relatives unaffected by cancer to give 7.26 relatives per proband on average.

[4]The percentage of variants detected is calculated using the distribution of genotype totals above, for example 1109/1504 *BRCA1* (73.74%).

Abbreviations: IQR, interquartile range; P/LP, pathogenic/likely pathogenic

Table S12. Clinical outcomes in probands and relatives for the sensitivity analysis

Group	Outcome	Scenario 3: Optimised referral of breast and ovarian cancer		Scenario 4: Genetic testing all breast cancers	
		**Mean**	**95% CI**	**Mean**	**95% CI**
**Probands with a P/LP and their relatives[1]**	Life expectancy	85.652	(85.516, 85.789)	85.958	(85.825, 86.091)
	Life years saved	42.118	(41.787, 42.449)	41.887	(41.519, 42.255)
**Relatives[1] with a P/LP variant only**	Life expectancy	83.973	(83.667, 84.279)	84.159	(83.818, 84.501)
	Life years saved	49.150	(48.595, 49.705)	49.112	(48.495, 49.730)
	Bilateral risk-reducing mastectomy uptake, n (%)	1181 (16.9%)		2747 (39.6%)	
	Risk-reducing salpingo-oophorectomy uptake, n (%)	2739 (39.2%)		6166 (89.0%)	
	Breast cancer incidence, n (%)	2777 (39.7%)		2055 (29.7%)	
	Ovarian cancer incidence, n (%)	485 (6.9%)		141 (2.0%)	

Full P/LP variant detection following a cancer diagnosis was limited to the probands only. Relatives of probands identified as P/LP variant carriers were assumed to undergo predictive testing within one year at a rate of 100%.

[1]Included relatives were female, and unaffected by cancer at the time of the proband’s breast cancer diagnosis.

Abbreviations: CI, confidence interval; P/LP, pathogenic/likely pathogenic

References

1. Sokolova A, Johnstone KJ, McCart Reed AE, Simpson PT, Lakhani SR. Hereditary breast cancer: syndromes, tumour pathology and molecular testing. Histopathology. 2023 Jan;82(1):70–82.

2. Nickson C, Smith MA, Feletto E, Velentzis LS, Broun K, Deij S, et al. A modelled evaluation of the impact of COVID-19 on breast, bowel, and cervical cancer screening programmes in Australia. Elife. 2023 Apr 6;12:e82818.

3. Sopik V, Narod SA. The relationship between tumour size, nodal status and distant metastases: on the origins of breast cancer. Breast Cancer Res Treat. 2018 Aug 1;170(3):647–56.

4. Rowley SM, Mascarenhas L, Devereux L, Li N, Amarasinghe KC, Zethoven M, et al. Population-based genetic testing of asymptomatic women for breast and ovarian cancer susceptibility. Genet Med. 2019 Apr;21(4):913–22.

5. Norquist BM, Harrell MI, Brady MF, Walsh T, Lee MK, Gulsuner S, et al. Inherited mutations in women with ovarian carcinoma. JAMA Oncol. 2016 Apr;2(4):482–90.

6. Kuchenbaecker KB, Hopper JL, Barnes DR, Phillips KA, Mooij TM, Roos-Blom MJ, et al. Risks of breast, ovarian, and contralateral breast cancer for *BRCA1* and *BRCA2* mutation carriers. JAMA. 2017 Jun 20;317(23):2402–16.

7. Yang X, Leslie G, Doroszuk A, Schneider S, Allen J, Decker B, et al. Cancer risks associated with germline *PALB2* pathogenic variants: an international study of 524 families. J Clin Oncol. 2020 Mar 1;38(7):674–85.

8. Schmidt MK, Hogervorst F, van Hien R, Cornelissen S, Broeks A, Adank MA, et al. Age- and tumor subtype-specific breast cancer risk estimates for *CHEK2**1100delC carriers. J Clin Oncol. 2016 Aug 10;34(23):2750–60.

9. Couch FJ, Shimelis H, Hu C, Hart SN, Polley EC, Na J, et al. Associations between cancer predisposition testing panel genes and breast cancer. JAMA Oncol. 2017 Sep 1;3(9):1190.

10. Yang X, Song H, Leslie G, Engel C, Hahnen E, Auber B, et al. Ovarian and breast cancer risks associated with pathogenic variants in *RAD51C* and *RAD51D*. J Natl Cancer Inst. 2020 Dec 14;112(12):1242–50.

11. Ramus SJ, Song H, Dicks E, Tyrer JP, Rosenthal AN, Intermaggio MP, et al. Germline mutations in the *BRIP1*, *BARD1*, *PALB2*, and *NBN* genes in women with ovarian cancer. J Natl Cancer Inst. 2015 Nov;107(11).

12. Mavaddat N, Michailidou K, Dennis J, Lush M, Fachal L, Lee A, et al. Polygenic risk scores for prediction of breast cancer and breast cancer subtypes. Am J Hum Genet. 2019 Jan 3;104(1):21–34.

13. Yang X, Leslie G, Gentry-Maharaj A, Ryan A, Intermaggio M, Lee A, et al. Evaluation of polygenic risk scores for ovarian cancer risk prediction in a prospective cohort study. J Med Genet. 2018 Aug;55(8):546–54.

14. Surveillance E and End Results (SEER) Program. SEER*Stat Database: Incidence - SEER 18 Regs Research Data (1992-2014) [Internet]. National Cancer Institute; 2017. Available from: www.seer.cancer.gov

15. Petelin L, Hossack L, Mitchell G, Liew D, Trainer AH, James PA. A microsimulation model for evaluating the effectiveness of cancer risk management for BRCA pathogenic variant carriers: miBRovaCAre. Value Health. 2019 Aug;22(8):854–62.

16. Candido-dos-Reis FJ, Song H, Goode EL, Cunningham JM, Fridley BL, Larson MC, et al. Germline min *BRCA1* or *BRCA2* and ten-sear survival for women diagnosed with epithelial ovarian cancer. Clin Cancer Res. 2015 Feb 1;21(3):652–7.

17. Australian Institute of Health and Welfare. BreastScreen Australia monitoring report 2018. Canberra, Australia; 2018. (Cancer Series No 112). Report No.: Cat no CAN116.

18. Vreemann S, Gubern-Merida A, Schlooz-Vries MS, Bult P, van Gils CH, Hoogerbrugge N, et al. Influence of risk category and screening round on the performance of an MR imaging and mammography screening program in carriers of the *BRCA* mutation and other women at increased risk. Radiology. 2018 Feb;286(2):443–51.

19. Australian Bureau of Statistics. Fertility, by age, by state [Internet]. Canberra; 2019 [accessed 2019 May 21]. Available from: https://stat.data.abs.gov.au

20. Wong-Brown MW, Meldrum CJ, Carpenter JE, Clarke CL, Narod SA, Jakubowska A, et al. Prevalence of *BRCA1* and *BRCA2* germline mutations in patients with triple-negative breast cancer. Breast Cancer Res Treat. 2015 Feb;150(1):71–80.

21. Alsop K, Fereday S, Meldrum C, deFazio A, Emmanuel C, George J, et al. *BRCA* mutation frequency and patterns of treatment response in *BRCA* mutation–positive women with ovarian cancer: a report from the Australian Ovarian Cancer Study group. J Clin Oncol. 2012 Jul 20;30(21):2654–63.

22. Australian Institute of Health and Welfare. Ovarian cancer in Australia: an overview, 2010 [Internet]. Canberra; [accessed 2020 Jan 29]. Available from: https://www.aihw.gov.au/reports-data?id=6442468331

23. Farshid G, Walters D. Molecular subtypes of screen-detected breast cancer. Breast Cancer Res Treat. 2018 Nov 1;172(1):191–9.

24. Australian Institute of Health and Welfare. Australian Cancer Database [Internet]. Canberra; [accessed 2020 Jul 10]. Available from: https://www.aihw.gov.au/about-our-data/our-data-collections/australian-cancer-database

25. Kentwell M, Dow E, Antill Y, Wrede CD, McNally O, Higgs E, et al. Mainstreaming cancer genetics: A model integrating germline *BRCA* testing into routine ovarian cancer clinics. Gynecol Oncol. 2017 Apr;145(1):130–6.

26. Crispin M, Udovicich C, Chan S, Wong S, Pitcher M. Trends in genetic screening referral in breast cancer patients under the age of forty: 2001-2016. Breast J. 2018 Nov;24(6):1109–11.
